# Aryl derivatives of 3*H*-1,2-benzoxaphosphepine 2-oxides as inhibitors of cancer-related carbonic anhydrase isoforms IX and XII

**DOI:** 10.1080/14756366.2023.2249267

**Published:** 2023-09-01

**Authors:** Anastasija Balašova, Aleksandrs Pustenko, Alessio Nocentini, Daniela Vullo, Claudiu T. Supuran, Raivis Žalubovskis

**Affiliations:** a Latvian Institute of Organic Synthesis, Riga, Latvia; b Institute of Technology of Organic Chemistry, Riga Technical University, Riga, Latvia; c Department of Neurofarba, Section of Pharmaceutical and Nutraceutical Sciences, Florence, Italy

**Keywords:** Carbonic anhydrase, benzoxaphosphepine 2-oxide, isoform-selective inhibitors, anticancer, Suzuki reaction

## Abstract

A range of 3*H*-1,2-benzoxaphosphepine 2-oxide aryl derivatives with various substitution patterns at positions 7, 8, or 9 of the scaffold was synthesised in five steps from the commercially available salicylaldehydes. All of the newly obtained compounds were studied for their inhibition potency against carbonic anhydrase (CA) isoforms I, II, IX, and XII. Delightfully, these compounds showed a striking selectivity for the cancer-associated CA IX and XII over the cytosolic CA I and II, whose inhibition may lead to side-effects. Overall, a structure–activity relationship (SAR) revealed that 7- and 8-substituted aryl derivatives were more effective inhibitors of CA IX and XII than 9-substituted derivatives. In addition, the fluorine-containing analogues emerged as the most potent CA IX/XII inhibitors in this series.

## Graphical Abstract

**Figure:**
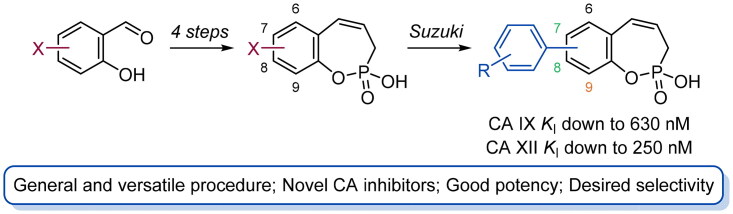

## Introduction

Cancer is a devastating group of diseases which is one of the major causes of death worldwide, accounting for nearly 10 million deaths in 2020^1^. By approaching to the middle of the 21st century, the cancer incidence and mortality rates are expected to increase to 29.5 million and 16.4 million per year, respectively^2^. The development of novel and improved therapies to combat cancer is therefore of a paramount priority. Carbonic anhydrase (CA, EC 4.2.1.1) isoforms CA IX and CA XII are presently serving as biomarkers and anticancer drug targets^3^. Both of these isozymes are highly overexpressed in various cancer types and may contribute to the growth of cancer, subsequent metastasis, as well as impaired therapeutic response^4^.

CA IX and CA XII are transmembrane zinc metalloenzymes that belong to the *α*-CA family^5^. Humans have 15 *α*-CA isoforms with different expression patterns, molecular features and kinetic properties^6^. These enzymes are involved in many important physiological processes (e.g. respiration, homeostasis, and metabolism), as they catalyse the reversible hydration of CO_2_^6^. Hence, the development of selective CA IX and XII inhibitors is highly desired to prevent possible side effects.

At present, none of the clinically used CA inhibitors displays selectivity for a specific isoform^7^. Due to the high level of structural homology between the CA isoforms and sequence similarities within the active site, the design and development of isoform-specific CA inhibitors remain challenging^8^. However, to our knowledge, to date, several molecules have been reported as potent and selective CA IX and XII inhibitors, including coumarins^9–12^, isocoumarins^13^, thiocoumarins^9^^,^^12^, sulfocoumarins^9^^,^^14–17^, and their congeners—homosulfocoumarins^18^ (3*H*-1,2-benzoxathiepine 2,2-dioxides).

In the course of our research, devoted to discovering novel chemotypes acting as selective CA IX/XII inhibitors that could be employed in cancer chemotherapy, we previously designed and synthesised a series of 3*H*-1,2-benzoxaphosphepine 2-oxides as bioisosteres of homosulfocoumarins^19^. These new compounds showed excellent selectivity and good inhibitory activity against both CA IX and XII. Our interest in phosphorus heterocycles stems from the fact that phosphorus functionalities can improve the pharmacokinetics profile, bioavailability and water solubility of drugs^20^. Moreover, several groups reported the use of organophosphorus compounds as CA inhibitors^21^.

In continuation of our previous work on the development of benzoxaphosphepine 2-oxides as CA inhibitors^19^, the current study is aimed to investigate the chemical space around this novel chemotype. In this paper, we present the synthesis and biological evaluation of 7-, 8- and 9-aryl-substituted benzoxaphosphepine 2-oxides.

## Materials and methods

### Chemistry

The air- or moisture-sensitive reactions were performed under an argon atmosphere using dry glassware. All reagents, starting materials and solvents were purchased from commercial sources and used as received. TLC was performed on silica gel plates (60 F_254_) and visualised under UV light (254 and 365 nm). Reversed-phase chromatography was done on a Biotage Isolera One system using Biotage SNAP KP-C18-HS cartridges. Melting points were determined on an OptiMelt MPA100 apparatus. IR spectra were recorded on a Shimadzu FTIR IR Prestige-21 spectrophotometer. ^1^H, ^13^C and ^31^P NMR spectra were recorded on a Bruker Avance Neo 400 MHz spectrometer. The chemical shifts (*δ*) were reported in parts per million (ppm) relative to the residual solvent peak as an internal reference (DMSO-*d*_6_: ^1^H 2.50, ^13^C 39.52; CD_3_OD: ^1^H 3.31, ^13^C 49.00). ^31^P shifts were referenced externally to H_3_PO_4_. The coupling constants (*J*) were expressed in Hertz (Hz). HRMS was performed on a Q-TOF Micro mass spectrometer.

The synthesis and characterisation of 4-iodo-2-vinylphenol (**2a**), 2-bromo-6-vinylphenol (**2c**), ethyl allylphosphonochloridate (**3**), ethyl (4-iodo-2-vinylphenyl) allylphosphonate (**4a**), 2-bromo-6-vinylphenyl ethyl allylphosphonate (**4c**) together with corresponding 3*H*-1,2-benzoxaphosphepine 2-oxides **6a,c** and **7a,c** are reported by our group in the previous paper^19^.

#### 5-Bromo-2-vinylphenol (2b)

The titled compound **2b** was obtained according to the general procedure previously reported^19^ using MePPh_3_Br (16.35 g, 45.77 mmol), *t*BuOK (5.25 g, 46.8 mmol), and 4-bromo-2-hydroxybenzaldehyde (4.00 g, 19.9 mmol) as a yellowish solid (3.21 g, 81%). The NMR spectra are consistent with the literature^19^. ^1^H NMR (400 MHz, DMSO-*d_6_*) *δ* = 5.24 (dd, 1H, *J* = 11.3, 1.6 Hz), 5.79 (dd, 1H, *J* = 17.6, 1.6 Hz), 6.87 (dd, 1H, *J* = 17.6, 11.3 Hz), 6.95 (dd, 1H, *J* = 8.3, 2.0 Hz), 7.00 (d, 1H, *J* = 2.0 Hz), 7.37 (d, 1H, *J* = 8.3 Hz), and 10.13 (s, 1H) ppm. ^13^C NMR (101 MHz, DMSO-*d_6_*) *δ* = 114.6, 118.3, 120.8, 122.0, 123.5, 128.0, 130.8, and 155.7 ppm.

#### 5-Bromo-2-vinylphenyl ethyl allylphosphonate (4b)

The titled compound **4b** was obtained according to the general procedure previously reported^19^ using 5-bromo-2-vinylphenol (**2b**) (3.87 g, 19.4 mmol), ethyl allylphosphonochloridate (**3**) (3.46 ml, 23.3 mmol) and NEt_3_ (3.38 ml, 24.3 mmol) as a colourless oil (4.23 g, 66%). IR (thin film, cm^−1^): 1265 (P = O), 1218 (P = O), 1181 (P = O). ^31^P NMR (162 MHz, DMSO-*d_6_*) *δ* = 25.50 ppm. ^1^H NMR (400 MHz, DMSO-*d_6_*) *δ* = 1.20 (t, 3H, *J* = 7.0 Hz), 2.94 (dt, 1H, *J* = 7.3, 1.2 Hz), 2.99 (dt, 1H, *J* = 7.3, 1.2 Hz), 4.02–4.18 (m, 2H), 5.20–5.33 (m, 2H), 5.42 (dd, 1H, *J* = 11.2, 1.0 Hz), 5.70–5.83 (m, 1H), 5.91 (dd, 1H, *J* = 16.6, 1.0 Hz), 6.84–6.93 (m, 1H), 7.38–7.43 (m, 1H), 7.48–7.50 (m, 1H), and 7.62–7.66 (m, 1H) ppm. ^13^C NMR (101 MHz, DMSO-*d_6_*) *δ* = 16.1 (d, *J*_P,C_ = 5.6 Hz), 30.9 (d, *J*_P,C_ = 137 Hz), 62.8 (d, *J*_P,C_ = 6.8 Hz), 117.3, 120.5 (d, *J*_P,C_ = 1.4 Hz), 120.6 (d, *J*_P,C_ = 15.0 Hz), 123.7 (d, *J*_P,C_ = 2.6 Hz), 127.3 (d, *J*_P,C_ = 11.6 Hz), 128.0, 128.2 (d, *J*_P,C_ = 5.0 Hz), 129.2, and 147.8 (d, *J*_P,C_ = 9.1 Hz) ppm. HRMS (ESI) [M + H]^+^: *m/z* calcd for C_13_H_17_O_3_PBr: 331.0099, found 331.0114.

#### 8-Bromo-2-ethoxy-3H-benzo[f][1,2]oxaphosphepine 2-oxide (6b)

The titled compound **6b** was obtained according to the general procedure previously reported^19^ using 5-bromo-2-vinylphenyl ethyl allylphosphonate (**4b**) (2.00 g, 6.04 mmol) and ruthenium catalyst **5** (CAS: 250220–36-1) (286 mg, 0.30 mmol) as a greenish dense oil (1.51 g, 83%). IR (thin film, cm^−1^): 1270 (P = O), 1237 (P = O), 1202 (P = O). ^31^P NMR (162 MHz, DMSO-*d_6_*) *δ* = 39.41 ppm. ^1^H NMR (400 MHz, DMSO-*d_6_*) *δ* = 1.28 (t, 3H, *J* = 7.0 Hz), 2.68–2.93 (m, 2H), 4.15–4.25 (m, 2H), 5.94–6.04 (m, 1H), 6.66–6.70 (m, 1H), 7.28–7.32 (m, 1H), 7.43–7.48 (m, 2H) ppm. ^13^C NMR (101 MHz, DMSO-*d_6_*) *δ* = 16.2 (d, *J*_P,C_ = 5.7 Hz), 25.6 (d, *J*_P,C_ = 125 Hz), 62.5 (d, *J*_P,C_ = 6.8 Hz), 121.3, 123.3 (d, *J*_P,C_ = 12.2 Hz), 124.4 (d, *J*_P,C_ = 3.5 Hz), 127.1, 128.0, 128.7 (d, *J*_P,C_ = 8.8 Hz), 132.3, and 147.9 (d, *J*_P,C_ = 7.8 Hz) ppm. HRMS (ESI) [M + H]^+^: *m/z* calcd for C_11_H_13_O_3_PBr: 302.9786, found 302.9781.

#### 8-Bromo-2-hydroxy-3H-benzo[f][1,2]oxaphosphepine 2-oxide (7b)

The titled compound **7b** was obtained according to the general procedure previously reported^19^ using 8-bromo-2-ethoxy-3*H*-benzo[*f*][1,2]oxaphosphepine 2-oxide (**6b**) (1.13 g, 3.73 mmol) and TMSBr (2.93 ml, 22.4 mmol) as a white solid (0.88 g, 86%). Mp: 182–184 °C. IR (KBr, cm^−1^): 2519 (O = P-OH), 2245 (O = P-OH), 1250 (P = O), and 1205 (P = O). ^31^P NMR (162 MHz, DMSO-*d_6_*) *δ* = 35.97 ppm. ^1^H NMR (400 MHz, DMSO-*d_6_*) *δ* = 2.58–2.68 (m, 2H), 5.91–6.04 (m, 1H), 6.57–6.63 (m, 1H), 7.24–7.29 (m, 1H), 7.31–7.34 (m, 1H), 7.38–7.42 (m, 1H) ppm. ^13^C NMR (101 MHz, DMSO-*d_6_*) *δ* = 27.3 (d, *J*_P,C_ = 125 Hz), 120.9, 124.4 (d, *J*_P,C_ = 12.0 Hz), 124.6 (d, *J*_P,C_ = 3.2 Hz), 127.5, 128.2 (d, *J*_P,C_ = 8.4 Hz), 132.2, and 148.5 (d, *J*_P,C_ = 7.4 Hz) ppm. HRMS (ESI) [M–H]^–^: *m/z* calcd for C_9_H_7_O_3_PBr: 272.9316, found 272.9320.

### General procedure for the synthesis of 3H-1,2-benzoxaphosphepine 2-oxide aryl derivatives 8–10

The corresponding 3*H*-1,2-benzoxaphosphepine 2-oxide halogen derivative **7** (200 mg, 1.0 eq) was placed in a pressure tube and dissolved in 1,4-dioxane (5 ml) followed by the addition of degassed water (1 ml). The corresponding boronic acid (1.5 eq), K_2_CO_3_ (2.0 eq) and Pd(dppf)Cl_2_ (10 mol% for iodo derivative **7a**; 20 mol% in case of bromo derivatives **7b** and **7c**) were added to the solution. The reaction mixture was purged with argon for 5 min, the tube was sealed and heated for 16 h at 80 °C. Upon cooling to rt, the reaction mixture was filtered through a pad of celite, which was washed with MeCN. The pH of the filtrate was adjusted to 2 by addition of TFA. After that, the filtrate was concentrated *in vacuo*. The crude product was purified by reversed-phase flash chromatography (MeCN/water = 10 to 95%) and recrystallised from EtOAc.

#### 2-Hydroxy-7-phenyl-3H-benzo[f][1,2]oxaphosphepine 2-oxide (8a)

By following the general procedure, **8a** was prepared from 2-hydroxy-7-iodo-3*H*-benzo[*f*][1,2]oxaphosphepine 2-oxide (**7a**) (200 mg, 0.62 mmol), phenylboronic acid (114 mg, 0.93 mmol), K_2_CO_3_ (172 mg, 1.24 mmol), and Pd(dppf)Cl_2_ (45 mg, 0.062 mmol) as a white solid (101 mg, 60%). Decomp. > 205 °C. IR (KBr, cm^−1^): 2611 (O = P-OH), 2161 (O = P-OH), 1619 (O = P-OH), 1215 (P = O), and 1196 (P = O). ^31^P NMR (162 MHz, DMSO-*d_6_*) *δ* = 35.77 ppm. ^1^H NMR (400 MHz, DMSO-*d_6_*) *δ* = 2.62 (d, 1H, *J* = 6.6 Hz), 2.67 (d, 1H, *J* = 6.6 Hz), 5.92–6.05 (m, 1H), 6.69–6.76 (m, 1H), 7.15–7.22 (m, 1H), 7.32–7.40 (m, 1H), 7.42–7.50 (m, 2H), and 7.56–7.70 (m, 4H) ppm. ^13^C NMR (101 MHz, DMSO-*d_6_*) *δ* = 27.3 (d, *J*_P,C_ = 125 Hz), 122.3 (d, *J*_P,C_ = 3.2 Hz), 124.0 (d, *J*_P,C_ = 12.2 Hz), 126.7, 127.5 (d, *J*_P,C_ = 2.4 Hz), 128.3, 128.8, 129.0, 129.1 (d, *J*_P,C_ = 8.6 Hz), 136.4, 139.2, and 147.6 (d, *J*_P,C_ = 7.5 Hz) ppm. HRMS (ESI) [M + H]^+^: *m/z* calcd for C_15_H_14_O_3_P: 273.0681, found 273.0685.

#### 2-Hydroxy-7–(4-methoxyphenyl)-3H-benzo[f][1,2]oxaphosphepine 2-oxide (8b)

By following the general procedure, **8b** was prepared from 2-hydroxy-7-iodo-3*H*-benzo[*f*][1,2]oxaphosphepine 2-oxide (**7a**) (200 mg, 0.62 mmol), (4-methoxyphenyl)boronic acid (142 mg, 0.93 mmol), K_2_CO_3_ (172 mg, 1.24 mmol), and Pd(dppf)Cl_2_ (45 mg, 0.062 mmol) as a white solid (115 mg, 61%). Mp: 214–216 °C. IR (KBr, cm^−1^): 2522 (O = P-OH), 2207 (O = P-OH), 1265 (P = O), and 1219 (P = O). ^31^P NMR (162 MHz, DMSO-*d_6_*) *δ* = 36.14 ppm. ^1^H NMR (400 MHz, DMSO-*d_6_*) *δ* = 2.61 (d, 1H, *J* = 6.1 Hz), 2.66 (d, 1H, *J* = 6.1 Hz), 3.79 (s, 3H), 5.91–6.04 (m, 1H), 6.67–6.74 (m, 1H), 6.98–7.05 (m, 2H), 7.13–7.19 (m, 1H), and 7.50–7.64 (m, 4H) ppm. ^13^C NMR (101 MHz, DMSO-*d_6_*) *δ* = 27.2 (d, *J*_P,C_ = 125 Hz), 55.2, 114.4, 122.2 (d, *J*_P,C_ = 3.2 Hz), 123.8 (d, *J*_P,C_ = 12.2 Hz), 126.9, 127.7, 128.1, 128.2, 129.2 (d, *J*_P,C_ = 8.5 Hz), 131.5, 136.1, 147.0 (d, *J*_P,C_ = 7.5 Hz), and 158.9 ppm. HRMS (ESI) [M + H]^+^: *m/z* calcd for C_16_H_16_O_4_P: 303.0786, found 303.0798.

#### Ethyl 4–(2-hydroxy-2-oxido-3H-benzo[f][1,2]oxaphosphepin-7-yl)benzoate (8c)

By following the general procedure, **8c** was prepared from 2-hydroxy-7-iodo-3*H*-benzo[*f*][1,2]oxaphosphepine 2-oxide (**7a**) (200 mg, 0.62 mmol), (4-(ethoxycarbonyl)phenyl)boronic acid (182 mg, 0.93 mmol), K_2_CO_3_ (172 mg, 1.24 mmol), and Pd(dppf)Cl_2_ (45 mg, 0.062 mmol) as a white solid (126 mg, 59%). Mp: 250–252 °C. IR (KBr, cm^−1^): 2512 (O = P-OH), 2187 (O = P-OH), 1710 (C = O), 1286 (P = O), and 1218 (P = O). ^31^P NMR (162 MHz, DMSO-*d_6_*) *δ* = 35.17 ppm. ^1^H NMR (400 MHz, DMSO-*d_6_*) *δ* = 1.34 (t, 3H, *J* = 7.0 Hz), 2.61 (d, 1H, *J* = 6.0 Hz), 2.66 (d, 1H, *J* = 6.0 Hz), 5.93–6.06 (m, 1H), 6.67–6.76 (m, 1H), 7.17–7.25 (m, 1H), 7.63–7.74 (m, 2H), 7.77–7.86 (m, 2H), and 7.97–8.07 (m, 2H) ppm. ^13^C NMR (101 MHz, DMSO-*d_6_*) *δ* = 14.2, 27.4 (d, *J*_P,C_ = 125 Hz), 60.8, 122.6 (d, *J*_P,C_ = 3.2 Hz), 124.3 (d, *J*_P,C_ = 12.2 Hz), 126.8, 127.7, 128.6, 128.7, 128.8 (d, *J*_P,C_ = 8.4 Hz), 129.2, 129.8, 134.9, 143.6, 148.4 (d, *J*_P,C_ = 7.6 Hz), and 165.5 ppm. HRMS (ESI) [M + H]^+^: *m/z* calcd for C_18_H_18_O_5_P: 345.0892, found 345.0901.

#### 7–(4-Fluorophenyl)-2-hydroxy-3H-benzo[f][1,2]oxaphosphepine 2-oxide (8d)

By following the general procedure, **8d** was prepared from 2-hydroxy-7-iodo-3*H*-benzo[*f*][1,2]oxaphosphepine 2-oxide (**7a**) (200 mg, 0.62 mmol), (4-fluorophenyl)boronic acid (130 mg, 0.93 mmol), K_2_CO_3_ (172 mg, 1.24 mmol), and Pd(dppf)Cl_2_ (45 mg, 0.062 mmol) as a white solid (114 mg, 63%). Decomp. > 232 °C. IR (KBr, cm^−1^): 2514 (O = P-OH), 2177 (O = P-OH), 1648 (O = P-OH), 1214 (P = O), and 1200 (P = O). ^31^P NMR (162 MHz, DMSO-*d_6_*) *δ* = 35.88 ppm. ^1^H NMR (400 MHz, DMSO-*d_6_*) *δ* = 2.59–2.70 (m, 2H), 5.91–6.06 (m, 1H), 6.67–6.75 (m, 1H), 7.15–7.22 (m, 1H), 7.24–7.33 (m, 2H), 7.55–7.63 (m, 2H), and 7.66–7.75 (m, 2H) ppm. ^13^C NMR (101 MHz, DMSO-*d_6_*) *δ* = 27.3 (d, *J*_P,C_ = 125 Hz), 115.7 (d, *J*_F,C_ = 21.0 Hz), 122.3 (d, *J*_P,C_ = 3.2 Hz), 124.0 (d, *J*_P,C_ = 12.2 Hz), 127.4, 128.3, 128.6 (d, *J*_F,C_ = 8.2 Hz), 128.8, 129.1 (d, *J*_P,C_ = 8.6 Hz), 135.4, 135.6 (d, *J*_F,C_ = 2.4 Hz), 147.5 (d, *J*_P,C_ = 7.4 Hz), and 162.0 (d, *J*_F,C_ = 244 Hz) ppm. HRMS (ESI) [M + H]^+^: *m/z* calcd for C_15_H_13_O_3_PF: 291.0586, found 291.0586.

#### 7–(4-Chlorophenyl)-2-hydroxy-3H-benzo[f][1,2]oxaphosphepine 2-oxide (8e)

By following the general procedure, **8e** was prepared from 2-hydroxy-7-iodo-3*H*-benzo[*f*][1,2]oxaphosphepine 2-oxide (**7a**) (200 mg, 0.62 mmol), (4-chlorophenyl)boronic acid (146 mg, 0.93 mmol), K_2_CO_3_ (172 mg, 1.24 mmol), and Pd(dppf)Cl_2_ (45 mg, 0.062 mmol) as a white solid (112 mg, 59%). Decomp. > 212 °C. IR (KBr, cm^−1^): 2089 (O = P-OH), 1637 (O = P-OH), 1201 (P = O), and 1117 (P = O). ^31^P NMR (162 MHz, DMSO-*d_6_*) *δ* = 35.74 ppm. ^1^H NMR (400 MHz, DMSO-*d_6_*) *δ* = 2.62 (d, 1H, *J* = 6.0 Hz), 2.67 (d, 1H, *J* = 6.0 Hz), 5.92–6.06 (m, 1H), 6.67–6.75 (m, 1H), 7.16–7.23 (m, 1H), 7.47–7.54 (m, 2H), and 7.58–7.73 (m, 4H) ppm. ^13^C NMR (101 MHz, DMSO-*d_6_*) *δ* = 27.3 (d, *J*_P,C_ = 125 Hz), 122.4 (d, *J*_P,C_ = 3.2 Hz), 124.1 (d, *J*_P,C_ = 12.2 Hz), 127.4, 128.4, 128.8, 128.9, 129.0 (d, *J*_P,C_ = 8.6 Hz), 132.4, 135.0, 137.9, and 147.8 (d, *J*_P,C_ = 7.5 Hz) ppm. HRMS (ESI) [M + H]^+^: *m/z* calcd for C_15_H_13_O_3_PCl: 307.0291, found 307.0290.

#### 2-Hydroxy-7–(4-(trifluoromethyl)phenyl)-3H-benzo[f][1,2]oxaphosphepine 2-oxide (8f)

By following the general procedure, **8f** was prepared from 2-hydroxy-7-iodo-3*H*-benzo[*f*][1,2]oxaphosphepine 2-oxide (**7a**) (200 mg, 0.62 mmol), (4-(trifluoromethyl)phenyl)boronic acid (177 mg, 0.93 mmol), K_2_CO_3_ (172 mg, 1.24 mmol), and Pd(dppf)Cl_2_ (45 mg, 0.062 mmol) as an off-white solid (133 mg, 63%). Decomp. > 280 °C. IR (KBr, cm^−1^): 1669 (O = P-OH), 1222 (P = O), and 1197 (P = O). ^31^P NMR (162 MHz, CD_3_OD) *δ* = 40.27 ppm. ^1^H NMR (400 MHz, CD_3_OD) *δ* = 2.52 (d, 1H, *J* = 6.7 Hz), 2.57 (d, 1H, *J* = 6.7 Hz), 5.98–6.11 (m, 1H), 6.63–6.69 (m, 1H), 7.23–7.27 (m, 1H), 7.47–7.50 (m, 1H), 7.52–7.57 (m, 1H), 7.60–7.63 (m, 2H), and 7.83–7.87 (m, 2H) ppm. ^13^C NMR (101 MHz, CD_3_OD) *δ* = 29.2 (d, *J*_P,C_ = 125 Hz), 121.7, 124.1 (d, *J*_P,C_ = 3.0 Hz), 124.3 (q, *J*_F,C_ = 3.6 Hz), 124.7 (q, *J*_F,C_ = 3.5 Hz), 126.5 (d, *J*_P,C_ = 12.2 Hz), 127.1, 128.4, 129.7 (d, *J*_P,C_ = 8.3 Hz), 129.9, 130.7, 131.6, 132.2 (q, *J*_F,C_ = 32.0 Hz), 136.3, 142.7, and 151.1 (d, *J*_P,C_ = 7.5 Hz) ppm. HRMS (ESI) [M + H]^+^: *m/z* calcd for C_16_H_13_O_3_PF_3_: 341.0554, found 341.0566.

#### 7–(3,5-Dichlorophenyl)-2-hydroxy-3H-benzo[f][1,2]oxaphosphepine 2-oxide (8 g)

By following the general procedure, **8 g** was prepared from 2-hydroxy-7-iodo-3*H*-benzo[*f*][1,2]oxaphosphepine 2-oxide (**7a**) (200 mg, 0.62 mmol), (3,5-dichlorophenyl)boronic acid (178 mg, 0.93 mmol), K_2_CO_3_ (172 mg, 1.24 mmol), and Pd(dppf)Cl_2_ (45 mg, 0.062 mmol) as a white solid (119 mg, 56%). Decomp. > 180 °C. IR (KBr, cm^−1^): 2508 (O = P-OH), 2255 (O = P-OH), 1230 (P = O), and 1206 (P = O). ^31^P NMR (162 MHz, DMSO-*d_6_*) *δ* = 34.87 ppm. ^1^H NMR (400 MHz, DMSO-*d_6_*) *δ* = 2.61 (d, 1H, *J* = 5.8 Hz), 2.66 (d, 1H, *J* = 5.8 Hz), 5.92–6.05 (m, 1H), 6.66–6.73 (m, 1H), 7.15–7.21 (m, 1H), 7.55–7.60 (m, 1H), and 7.66–7.78 (m, 4H) ppm. ^13^C NMR (101 MHz, DMSO-*d_6_*) *δ* = 27.5 (d, *J*_P,C_ = 125 Hz), 122.5 (d, *J*_P,C_ = 3.2 Hz), 124.3 (d, *J*_P,C_ = 12.2 Hz), 125.3, 126.8, 127.7, 128.6, 128.8 (d, *J*_P,C_ = 8.5 Hz), 129.4, 133.2, 134.7, 142.6, and 148.5 (d, *J*_P,C_ = 7.5 Hz) ppm. HRMS (ESI) [M + H]^+^: *m/z* calcd for C_15_H_12_O_3_PCl_2_: 340.9901, found 340.9906.

#### 7–(3-Fluorophenyl)-2-hydroxy-3H-benzo[f][1,2]oxaphosphepine 2-oxide (8h)

By following the general procedure, **8h** was prepared from 2-hydroxy-7-iodo-3*H*-benzo[*f*][1,2]oxaphosphepine 2-oxide (**7a**) (200 mg, 0.62 mmol), (3-fluorophenyl)boronic acid (130 mg, 0.93 mmol), K_2_CO_3_ (172 mg, 1.24 mmol), and Pd(dppf)Cl_2_ (45 mg, 0.062 mmol) as a white solid (180 mg, 60%). Mp: 171–173 °C. IR (KBr, cm^−1^): 2579 (O = P-OH), 2308 (O = P-OH), and 1190 (P = O). ^31^P NMR (162 MHz, DMSO-*d_6_*) *δ* = 35.68 ppm. ^1^H NMR (400 MHz, DMSO-*d_6_*) *δ* = 2.60–2.72 (m, 2H), 5.90–6.08 (m, 1H), 6.67–6.77 (m, 1H), 7.14–7.24 (m, 2H), 7.46–7.60 (m, 3H), and 7.62–7.70 (m, 2H) ppm. ^13^C NMR (101 MHz, DMSO-*d_6_*) *δ* = 27.3 (d, *J*_P,C_ = 125 Hz), 113.4 (d, *J*_F,C_ = 22.2 Hz), 114.2 (d, *J*_F,C_ = 21.0 Hz), 122.4 (d, *J*_P,C_ = 3.2 Hz), 122.7, 124.0 (d, *J*_P,C_ = 12.2 Hz), 127.6, 128.4, 129.0, 129.1, 130.9 (d, *J*_P,C_ = 8.6 Hz), 135.0, 141.6 (d, *J*_F,C_ = 7.8 Hz), 148.0 (d, *J*_P,C_ = 7.6 Hz), and 162.7 (d, *J*_F,C_ = 243 Hz) ppm. HRMS (ESI) [M + H]^+^: *m/z* calcd for C_15_H_13_O_3_PF: 291.0586, found 291.0588.

#### 2-Hydroxy-7-(o-tolyl)-3H-benzo[f][1,2]oxaphosphepine 2-oxide (8i)

By following the general procedure, **8i** was prepared from 2-hydroxy-7-iodo-3*H*-benzo[*f*][1,2]oxaphosphepine 2-oxide (**7a**) (200 mg, 0.62 mmol), *o*-tolylboronic acid (127 mg, 0.93 mmol), K_2_CO_3_ (172 mg, 1.24 mmol), and Pd(dppf)Cl_2_ (45 mg, 0.062 mmol) as a white solid (101 mg, 57%). Mp: 156–158 °C. IR (KBr, cm^−1^): 2585 (O = P-OH), 2303 (O = P-OH), 1623 (O = P-OH), and 1193 (P = O). ^31^P NMR (162 MHz, DMSO-*d_6_*) *δ* = 35.70 ppm. ^1^H NMR (400 MHz, DMSO-*d_6_*) *δ* = 2.23 (s, 3H), 2.63 (d, 1H, *J* = 6.6 Hz), 2.68 (d, 1H, *J* = 6.6 Hz), 5.90–6.03 (m, 1H), 6.64–6.74 (m, 1H), and 7.14–7.32 (m, 7H) ppm. ^13^C NMR (101 MHz, DMSO-*d_6_*) *δ* = 20.2, 27.3 (d, *J*_P,C_ = 125 Hz), 121.6 (d, *J*_P,C_ = 3.2 Hz), 123.8 (d, *J*_P,C_ = 12.2 Hz), 126.0, 127.5, 127.7, 129.1 (d, *J*_P,C_ = 8.6 Hz), 129.6, 129.8, 130.4, 131.0, 134.8, 137.4, 140.2, and 147.0 (d, *J*_P,C_ = 7.5 Hz) ppm. HRMS (ESI) [M + H]^+^: *m/z* calcd for C_16_H_16_O_3_P: 287.0837, found 287.0841.

#### Methyl 3–(2-hydroxy-2-oxido-3H-benzo[f][1,2]oxaphosphepin-7-yl)benzoate (8j)

By following the general procedure, **8j** was prepared from 2-hydroxy-7-iodo-3*H*-benzo[*f*][1,2]oxaphosphepine 2-oxide (**7a**) (200 mg, 0.62 mmol), (3-(methoxycarbonyl)phenyl)boronic acid (168 mg, 0.93 mmol), K_2_CO_3_ (172 mg, 1.24 mmol), and Pd(dppf)Cl_2_ (45 mg, 0.062 mmol) as a white solid (117 mg, 57%). Mp: 145–147 °C. IR (KBr, cm^−1^): 2303 (O = P-OH), 1718 (C = O), 1252 (P = O), and 1220 (P = O). ^31^P NMR (162 MHz, DMSO-*d_6_*) *δ* = 35.62 ppm. ^1^H NMR (400 MHz, DMSO-*d_6_*) *δ* = 2.62 (d, 1H, *J* = 6.6 Hz), 2.67 (d, 1H, *J* = 6.6 Hz), 3.89 (s, 3H), 5.93–6.06 (m, 1H), 6.71–6.76 (m, 1H), 7.19–7.24 (m, 1H), 7.59–7.68 (m, 3H), 7.93–7.97 (m, 2H), and 8.17–8.19 (m, 1H) ppm. ^13^C NMR (101 MHz, DMSO-*d_6_*) *δ* = 27.4 (d, *J*_P,C_ = 125 Hz), 52.3, 122.5 (d, *J*_P,C_ = 3.0 Hz), 124.2 (d, *J*_P,C_ = 12.2 Hz), 127.1, 127.6, 128.1, 128.6, 128.9, 129.0, 129.5, 130.4, 131.5, 135.2, 139.7, 148.0 (d, *J*_P,C_ = 7.6 Hz), and 166.1 ppm. HRMS (ESI) [M + H]^+^: *m/z* calcd for C_17_H_16_O_5_P: 331.0735, found 331.0734.

#### 2-Hydroxy-7–(3-nitrophenyl)-3H-benzo[f][1,2]oxaphosphepine 2-oxide (8k)

By following the general procedure, **8k** was prepared from 2-hydroxy-7-iodo-3*H*-benzo[*f*][1,2]oxaphosphepine 2-oxide (**7a**) (200 mg, 0.62 mmol), (3-nitrophenyl)boronic acid (156 mg, 0.93 mmol), K_2_CO_3_ (172 mg, 1.24 mmol), and Pd(dppf)Cl_2_ (45 mg, 0.062 mmol) as a yellow solid (120 mg, 61%). Mp: 231–233 °C. IR (KBr, cm^−1^): 2580 (O = P-OH), 1646 (O = P-OH), and 1214 (P = O). ^31^P NMR (162 MHz, DMSO-*d_6_*) *δ* = 35.39 ppm. ^1^H NMR (400 MHz, DMSO-*d_6_*) *δ* = 2.63 (d, 1H, *J* = 6.6 Hz), 2.68 (d, 1H, *J* = 6.6 Hz), 5.94–6.07 (m, 1H), 6.71–6.77 (m, 1H), 7.21–7.26 (m, 1H), 7.72–7.78 (m, 3H), 8.12–8.23 (m, 2H), and 8.43–8.46 (m, 1H) ppm. ^13^C NMR (101 MHz, DMSO-*d_6_*) *δ* = 27.3 (d, *J*_P,C_ = 125 Hz), 121.0, 122.1, 122.6 (d, *J*_P,C_ = 3.2 Hz), 124.3 (d, *J*_P,C_ = 12.2 Hz), 127.8, 128.6, 128.8 (d, *J*_P,C_ = 8.5 Hz), 129.3, 130.5, 133.2, 133.9, 140.7, and 148.3, 148.4 ppm. HRMS (ESI) [M + H]^+^: *m/z* calcd for C_15_H_13_NO_5_P: 318.0531, found 318.0537.

#### 7–(4-(tert-Butyl)phenyl)-2-hydroxy-3H-benzo[f][1,2]oxaphosphepine 2-oxide (8 l)

By following the general procedure, **8 l** was prepared from 2-hydroxy-7-iodo-3*H*-benzo[*f*][1,2]oxaphosphepine 2-oxide (**7a**) (200 mg, 0.62 mmol), (4-(*tert*-butyl)phenyl)boronic acid (166 mg, 0.93 mmol), K_2_CO_3_ (172 mg, 1.24 mmol), and Pd(dppf)Cl_2_ (45 mg, 0.062 mmol) as a white solid (110 mg, 54%). Decomp. > 210 °C. IR (KBr, cm^−1^): 2162 (O = P-OH), 1654 (O = P-OH), 1220 (P = O), and 1195 (P = O). ^31^P NMR (162 MHz, DMSO-*d_6_*) *δ* = 31.16 ppm. ^1^H NMR (400 MHz, DMSO-*d_6_*) *δ* = 1.31 (s, 9H), 2.41–2.47 (m, 1H), 5.83–5.98 (m, 1H), 6.53–6.63 (m, 1H), 7.01–7.11 (m, 1H), 7.41–7.51 (m, 4H), and 7.52–7.60 (m, 2H) ppm. ^13^C NMR (101 MHz, DMSO-*d_6_*) *δ* = 28.7 (d, *J*_P,C_ = 125 Hz), 31.1, 34.2, 122.5 (d, *J*_P,C_ = 2.8 Hz), 125.3 (d, *J*_P,C_ = 11.4 Hz), 125.6, 126.2, 126.7, 128.3, 128.4 (d, *J*_P,C_ = 8.4 Hz), 128.7, 135.3, 136.6, 148.6 (d, *J*_P,C_ = 7.4 Hz), and 149.6 ppm. HRMS (ESI) [M + H]^+^: *m/z* calcd for C_19_H_22_O_3_P: 329.1307, found 329.1307.

#### 2-Hydroxy-8-phenyl-3H-benzo[f][1,2]oxaphosphepine 2-oxide (9a)

By following the general procedure, **9a** was prepared from 8-bromo-2-hydroxy-3*H*-benzo[*f*][1,2]oxaphosphepine 2-oxide (**7b**) (200 mg, 0.73 mmol), phenylboronic acid (133 mg, 1.09 mmol), K_2_CO_3_ (201 mg, 1.45 mmol), and Pd(dppf)Cl_2_ (106 mg, 0.15 mmol) as a white solid (121 mg, 61%). Mp: 155–157 °C. IR (KBr, cm^−1^): 2582 (O = P-OH), 2162 (O = P-OH), 1255 (P = O), and 1184 (P = O). ^31^P NMR (162 MHz, DMSO-*d_6_*) *δ* = 34.14 ppm. ^1^H NMR (400 MHz, DMSO-*d_6_*) *δ* = 2.60 (d, 1H, *J* = 6.4 Hz), 2.65 (d, 1H, *J* = 6.4 Hz), 5.87–6.01 (m, 1H), 6.60–6.67 (m, 1H), 7.34–7.41 (m, 3H), 7.44–7.52 (m, 3H), and 7.67–7.72 (m, 2H) ppm. ^13^C NMR (101 MHz, DMSO-*d_6_*) *δ* = 27.7 (d, *J*_P,C_ = 125 Hz), 119.6 (d, *J*_P,C_ = 2.8 Hz), 122.4, 124.0 (d, *J*_P,C_ = 11.6 Hz), 126.6, 127.1, 128.0, 128.6 (d, *J*_P,C_ = 8.0 Hz), 129.0, 131.3, 138.7, 141.0, and 148.7 (d, *J*_P,C_ = 7.1 Hz) ppm. HRMS (ESI) [M–H]^–^: *m/z* calcd for C_15_H_12_O_3_P: 271.0524, found 271.0536.

#### 2-Hydroxy-8–(4-methoxyphenyl)-3H-benzo[f][1,2]oxaphosphepine 2-oxide (9b)

By following the general procedure, **9b** was prepared from 8-bromo-2-hydroxy-3*H*-benzo[*f*][1,2]oxaphosphepine 2-oxide (**7b**) (200 mg, 0.73 mmol), (4-methoxyphenyl)boronic acid (166 mg, 1.09 mmol), K_2_CO_3_ (201 mg, 1.45 mmol), and Pd(dppf)Cl_2_ (106 mg, 0.15 mmol) as a white solid (147 mg, 67%). Mp: 205–207 °C. IR (KBr, cm^−1^): 2556 (O = P-OH), 2305 (O = P-OH), 1248 (P = O), and 1183 (P = O). ^31^P NMR (162 MHz, DMSO-*d_6_*) *δ* = 34.96 ppm. ^1^H NMR (400 MHz, DMSO-*d_6_*) *δ* = 2.63 (d, 1H, *J* = 6.4 Hz), 2.68 (d, 1H, *J* = 6.4 Hz), 3.80 (s, 3H), 5.85–6.00 (m, 1H), 6.61–6.67 (m, 1H), 7.00–7.06 (m, 2H), 7.32–7.36 (m, 2H), 7.45–7.49 (m, 1H), and 7.63–7.69 (m, 2H) ppm. ^13^C NMR (101 MHz, DMSO-*d_6_*) *δ* = 27.4 (d, *J*_P,C_ = 125 Hz), 55.2, 114.5, 118.9 (d, *J*_P,C_ = 3.2 Hz), 122.1, 123.3 (d, *J*_P,C_ = 11.8 Hz), 126.2, 127.8, 128.8 (d, *J*_P,C_ = 8.2 Hz), 130.9, 131.3, 140.8, 148.4 (d, *J*_P,C_ = 7.2 Hz), and 159.3 ppm. HRMS (ESI) [M–H]^–^: *m/z* calcd for C_16_H_14_O_4_P: 301.0630, found 301.0641.

#### Ethyl 4–(2-hydroxy-2-oxido-3H-benzo[f][1,2]oxaphosphepin-8-yl)benzoate (9c)

By following the general procedure, **9c** was prepared from 8-bromo-2-hydroxy-3*H*-benzo[*f*][1,2]oxaphosphepine 2-oxide (**7b**) (200 mg, 0.73 mmol), (4-(ethoxycarbonyl)phenyl)boronic acid (212 mg, 1.09 mmol), K_2_CO_3_ (201 mg, 1.45 mmol), and Pd(dppf)Cl_2_ (106 mg, 0.15 mmol) as a white solid (145 mg, 58%). Mp: 189–191 °C. IR (KBr, cm^−1^): 2577 (O = P-OH), 2287 (O = P-OH), 1708 (C = O), 1283 (P = O), and 1192 (P = O). ^31^P NMR (162 MHz, DMSO-*d_6_*) *δ* = 35.08 ppm. ^1^H NMR (400 MHz, DMSO-*d_6_*) *δ* = 1.34 (t, 3H, *J* = 7.1 Hz), 2.65 (d, 1H, *J* = 6.2 Hz), 2.70 (d, 1H, *J* = 6.2 Hz), 4.34 (q, 2H, *J* = 7.1 Hz), 5.92–6.06 (m, 1H), 6.64–6.72 (m, 1H), 7.39–7.51 (m, 2H), 7.57–7.65 (m, 1H), 7.85–7.91 (m, 2H), and 8.01–8.06 (m, 2H) ppm. ^13^C NMR (101 MHz, DMSO-*d_6_*) *δ* = 14.2, 27.4 (d, *J*_P,C_ = 125 Hz), 60.8, 119.9 (d, *J*_P,C_ = 3.2 Hz), 122.9, 124.2 (d, *J*_P,C_ = 12.0 Hz), 126.9, 127.9, 128.6 (d, *J*_P,C_ = 8.2 Hz), 129.1, 129.9, 131.5, 139.6, 143.0, 148.5 (d, *J*_P,C_ = 7.2 Hz), and 165.5 ppm. HRMS (ESI) [M–H]^–^: *m/z* calcd for C_18_H_16_O_5_P: 343.0735, found 343.0750.

#### 8–(4-Chlorophenyl)-2-hydroxy-3H-benzo[f][1,2]oxaphosphepine 2-oxide (9d)

By following the general procedure, **9d** was prepared from 8-bromo-2-hydroxy-3*H*-benzo[*f*][1,2]oxaphosphepine 2-oxide (**7b**) (200 mg, 0.73 mmol), (4-chlorophenyl)boronic acid (171 mg, 1.09 mmol), K_2_CO_3_ (201 mg, 1.45 mmol), and Pd(dppf)Cl_2_ (106 mg, 0.15 mmol) as a white solid (134 mg, 60%). Mp: 203–205 °C. IR (KBr, cm^−1^): 2583 (O = P-OH), 2292 (O = P-OH), 1218 (P = O), and 1187 (P = O). ^31^P NMR (162 MHz, DMSO-*d_6_*) *δ* = 35.06 ppm. ^1^H NMR (400 MHz, DMSO-*d_6_*) *δ* = 2.64 (d, 1H, *J* = 6.4 Hz), 2.69 (d, 1H, *J* = 6.4 Hz), 5.90–6.03 (m, 1H), 6.63–6.70 (m, 1H), 7.36–7.42 (m, 2H), 7.50–7.55 (m, 3H), and 7.72–7.77 (m, 2H) ppm. ^13^C NMR (101 MHz, DMSO-*d_6_*) *δ* = 27.4 (d, *J*_P,C_ = 125 Hz), 119.6 (d, *J*_P,C_ = 3.2 Hz), 122.6, 123.9 (d, *J*_P,C_ = 12.0 Hz), 127.3, 128.4, 128.7 (d, *J*_P,C_ = 8.4 Hz), 129.0, 131.4, 132.9, 137.4, 139.7, and 148.4 (d, *J*_P,C_ = 7.2 Hz) ppm. HRMS (ESI) [M–H]^–^: *m/z* calcd for C_15_H_11_O_3_PCl: 305.0134, found 305.0143.

#### 8–(3,5-Dichlorophenyl)-2-hydroxy-3H-benzo[f][1,2]oxaphosphepine 2-oxide (9e)

By following the general procedure, **9e** was prepared from 8-bromo-2-hydroxy-3*H*-benzo[*f*][1,2]oxaphosphepine 2-oxide (**7b**) (200 mg, 0.73 mmol), (3,5-dichlorophenyl)boronic acid (208 mg, 1.09 mmol), K_2_CO_3_ (201 mg, 1.45 mmol), and Pd(dppf)Cl_2_ (106 mg, 0.15 mmol) as a white solid (141 mg, 57%). Mp: 136–138 °C. IR (KBr, cm^−1^): 2533 (O = P-OH), 2262 (O = P-OH), 1221 (P = O), and 1196 (P = O). ^31^P NMR (162 MHz, DMSO-*d_6_*) *δ* = 33.47 ppm. ^1^H NMR (400 MHz, DMSO-*d_6_*) *δ* = 2.59 (d, 1H, *J* = 6.4 Hz), 2.64 (d, 1H, *J* = 6.4 Hz), 5.90–6.03 (m, 1H), 6.60–6.66 (m, 1H), 7.35–7.39 (m, 1H), 7.46–7.49 (m, 1H), 7.54–7.61 (m, 2H), and 7.74–7.78 (m, 2H) ppm. ^13^C NMR (101 MHz, DMSO-*d_6_*) *δ* = 27.8 (d, *J*_P,C_ = 125 Hz), 120.2, 122.6, 124.7, 125.3, 127.2, 128.3, 128.4, 131.4, 134.7, 137.8, 142.2, and 148.8 ppm. HRMS (ESI) [M–H]^–^: *m/z* calcd for C_15_H_10_O_3_PCl_2_: 338.9745, found 338.9760.

#### 2-Hydroxy-8–(2-nitrophenyl)-3H-benzo[f][1,2]oxaphosphepine 2-oxide (9f)

By following the general procedure, **9f** was prepared from 8-bromo-2-hydroxy-3*H*-benzo[*f*][1,2]oxaphosphepine 2-oxide (**7b**) (200 mg, 0.73 mmol), (2-nitrophenyl)boronic acid (182 mg, 1.09 mmol), K_2_CO_3_ (201 mg, 1.45 mmol), and Pd(dppf)Cl_2_ (106 mg, 0.15 mmol) as a yellowish solid (159 mg, 69%). Mp: 218–220 °C. IR (KBr, cm^−1^): 2604 (O = P-OH), 2240 (O = P-OH), 1185 (P = O), and 1126 (P = O). ^31^P NMR (162 MHz, DMSO-*d_6_*) *δ* = 34.84 ppm. ^1^H NMR (400 MHz, DMSO-*d_6_*) *δ* = 2.65 (d, 1H, *J* = 6.4 Hz), 2.70 (d, 1H, *J* = 6.4 Hz), 5.92–6.05 (m, 1H), 6.64–6.70 (m, 1H), 7.07–7.10 (m, 1H), 7.16 (dd, 1H, *J* = 8.0, 1.6 Hz), 7.38 (d, 1H, *J* = 8.0 Hz), 7.57–7.61 (m, 1H), 7.62–7.68 (m, 1H), 7.75–7.81 (m, 1H), and 8.01 (dd, 1H, *J* = 8.0, 1.0 Hz) ppm. ^13^C NMR (101 MHz, DMSO-*d_6_*) *δ* = 27.4 (d, *J*_P,C_ = 125 Hz), 120.9 (d, *J*_P,C_ = 3.3 Hz), 123.9, 124.2 (d, *J*_P,C_ = 11.6 Hz), 124.3, 127.7, 128.6 (d, *J*_P,C_ = 8.6 Hz), 129.3, 131.3, 131.8, 133.1, 133.7, 137.8, 148.0 (d, *J*_P,C_ = 7.2 Hz), and 148.7 ppm. HRMS (ESI) [M–H]^–^: *m/z* calcd for C_15_H_11_NO_5_P: 316.0375, found 316.0384.

#### 2-Hydroxy-9-phenyl-3H-benzo[f][1,2]oxaphosphepine 2-oxide (10a)

By following the general procedure, **10a** was prepared from 9-bromo-2-hydroxy-3*H*-benzo[*f*][1,2]oxaphosphepine 2-oxide (**7c**) (200 mg, 0.73 mmol), phenylboronic acid (133 mg, 1.09 mmol), K_2_CO_3_ (201 mg, 1.45 mmol), and Pd(dppf)Cl_2_ (106 mg, 0.15 mmol) as a white solid (166 mg, 84%). Mp: 184–186 °C. IR (KBr, cm^−1^): 2592 (O = P-OH), 2261 (O = P-OH), 1255 (P = O), and 1204 (P = O). ^31^P NMR (162 MHz, DMSO-*d_6_*) *δ* = 33.69 ppm. ^1^H NMR (400 MHz, DMSO-*d_6_*) *δ* = 2.67 (dd, 1H, *J* = 6.7, 1.0 Hz), 2.72 (dd, 1H, *J* = 6.7, 1.0 Hz), 5.86–5.99 (m, 1H), 6.62–6.68 (m, 1H), 7.23–7.39 (m, 4H), 7.40–7.46 (m, 2H), and 7.60–7.64 (m, 2H) ppm. ^13^C NMR (101 MHz, DMSO-*d_6_*) *δ* = 27.8 (d, *J*_P,C_ = 125 Hz), 123.3 (d, *J*_P,C_ = 11.6 Hz), 124.4, 127.2, 128.0, 128.5, 129.3 (d, *J*_P,C_ = 8.6 Hz), 129.6, 130.5 (d, *J*_P,C_ = 4.6 Hz), 134.3 (d, *J*_P,C_ = 3.4 Hz), 137.4, and 144.8 (d, *J*_P,C_ = 7.4 Hz) ppm. HRMS (ESI) [M–H]^–^: *m/z* calcd for C_15_H_12_O_3_P: 271.0524, found 271.0527.

#### 2-Hydroxy-9–(4-methoxyphenyl)-3H-benzo[f][1,2]oxaphosphepine 2-oxide (10b)

By following the general procedure, **10b** was prepared from 9-bromo-2-hydroxy-3*H*-benzo[*f*][1,2]oxaphosphepine 2-oxide (**7c**) (200 mg, 0.73 mmol), (4-methoxyphenyl)boronic acid (166 mg, 1.09 mmol), K_2_CO_3_ (201 mg, 1.45 mmol), and Pd(dppf)Cl_2_ (106 mg, 0.15 mmol) as a white solid (196 mg, 89%). Mp: 199–201 °C. IR (KBr, cm^−1^): 2573 (O = P-OH), 2257 (O = P-OH), 1248 (P = O), and 1205 (P = O). ^31^P NMR (162 MHz, DMSO-*d_6_*) *δ* = 34.24 ppm. ^1^H NMR (400 MHz, DMSO-*d_6_*) *δ* = 2.66 (d, 1H, *J* = 6.4 Hz), 2.71 (d, 1H, *J* = 6.4 Hz), 3.80 (s, 3H), 5.85–6.00 (m, 1H), 6.61–6.67 (m, 1H), 6.95–7.02 (m, 2H), 7.20–7.34 (m, 3H), and 7.54–7.60 (m, 2H) ppm. ^13^C NMR (101 MHz, DMSO-*d_6_*) *δ* = 27.6 (d, *J*_P,C_ = 125 Hz), 55.1, 113.5, 123.3 (d, *J*_P,C_ = 11.3 Hz), 124.4, 128.4, 129.4 (d, *J*_P,C_ = 7.8 Hz), 129.7, 130.0, 130.4, 130.8, 134.0, 144.8 (d, *J*_P,C_ = 7.4 Hz), and 158.6 ppm. HRMS (ESI) [M–H]^–^: *m/z* calcd for C_16_H_14_O_4_P: 301.0630, found 301.0636.

#### Ethyl 4–(2-hydroxy-2-oxido-3H-benzo[f][1,2]oxaphosphepin-9-yl)benzoate (10c)

By following the general procedure, **10c** was prepared from 9-bromo-2-hydroxy-3*H*-benzo[*f*][1,2]oxaphosphepine 2-oxide (**7c**) (200 mg, 0.73 mmol), (4-(ethoxycarbonyl)phenyl)boronic acid (212 mg, 1.09 mmol), K_2_CO_3_ (201 mg, 1.45 mmol), and Pd(dppf)Cl_2_ (106 mg, 0.15 mmol) as a white solid (208 mg, 83%). Mp: 194–196 °C. IR (KBr, cm^−1^): 2566 (O = P-OH), 2272 (O = P-OH), 1710 (C = O), 1285 (P = O), and 1190 (P = O). ^31^P NMR (162 MHz, DMSO-*d_6_*) *δ* = 34.39 ppm. ^1^H NMR (400 MHz, DMSO-*d_6_*) *δ* = 1.34 (t, 3H, *J* = 7.0 Hz), 2.66 (d, 1H, *J* = 6.1 Hz), 2.71 (d, 1H, *J* = 6.1 Hz), 4.35 (q, 2H, *J* = 7.0 Hz), 5.88–6.02 (m, 1H), 6.64–6.70 (m, 1H), 7.25–7.42 (m, 3H), 7.72–7.78 (m, 2H), and 7.98–8.03 (m, 2H) ppm. ^13^C NMR (101 MHz, DMSO-*d_6_*) *δ* = 14.2, 27.6 (d, *J*_P,C_ = 125 Hz), 60.8, 123.6 (d, *J*_P,C_ = 11.6 Hz), 124.6, 128.6, 128.8, 129.1 (d, *J*_P,C_ = 8.4 Hz), 130.0, 130.3, 131.2, 133.2 (d, *J*_P,C_ = 3.4 Hz), 142.2, 144.8 (d, *J*_P,C_ = 7.4 Hz), and 165.7 ppm. HRMS (ESI) [M–H]^–^: *m/z* calcd for C_18_H_16_O_5_P: 343.0735, found 343.0740.

#### 9–(4-Chlorophenyl)-2-hydroxy-3H-benzo[f][1,2]oxaphosphepine 2-oxide (10d)

By following the general procedure, **10d** was prepared from 9-bromo-2-hydroxy-3*H*-benzo[*f*][1,2]oxaphosphepine 2-oxide (**7c**) (200 mg, 0.73 mmol), (4-chlorophenyl)boronic acid (171 mg, 1.09 mmol), K_2_CO_3_ (201 mg, 1.45 mmol), and Pd(dppf)Cl_2_ (106 mg, 0.15 mmol) as a white solid (161 mg, 72%). Mp: 217–219 °C. IR (KBr, cm^−1^): 2568 (O = P-OH), 2261 (O = P-OH), 1256 (P = O), 1205 (P = O), and 1190 (P = O). ^31^P NMR (162 MHz, DMSO-*d_6_*) *δ* = 37.90 ppm. ^1^H NMR (400 MHz, DMSO-*d_6_*) *δ* = 2.61 (d, 1H, *J* = 6.2 Hz), 2.66 (d, *J* = 6.2 Hz), 5.84–5.97 (m, 1H), 6.58–6.65 (m, 1H), 7.21–7.27 (m, 1H), 7.28–7.35 (m, 2H), 7.44–7.50 (m, 2H), and 7.62–7.68 (m, 2H) ppm. ^13^C NMR (101 MHz, DMSO-*d_6_*) *δ* = 28.0 (d, *J*_P,C_ = 125 Hz), 123.9 (d, *J*_P,C_ = 10.4 Hz), 124.3, 127.5, 128.0, 128.7, 129.0 (d, *J*_P,C_ = 8.2 Hz), 130.2, 130.8, 131.5, 133.0 (d, *J*_P,C_ = 2.4 Hz), 136.0, and 145.1 (d, *J*_P,C_ = 7.4 Hz) ppm. HRMS (ESI) [M–H]^–^: *m/z* calcd for C_15_H_11_O_3_PCl: 305.0134, found 305.0143.

#### 9–(3,5-Dichlorophenyl)-2-hydroxy-3H-benzo[f][1,2]oxaphosphepine 2-oxide (10e)

By following the general procedure, **10e** was prepared from 9-bromo-2-hydroxy-3*H*-benzo[*f*][1,2]oxaphosphepine 2-oxide (**7c**) (200 mg, 0.73 mmol), (3,5-dichlorophenyl)boronic acid (208 mg, 1.09 mmol), K_2_CO_3_ (201 mg, 1.45 mmol), and Pd(dppf)Cl_2_ (106 mg, 0.15 mmol) as a white solid (164 mg, 66%). Mp: 210–212 °C. IR (KBr, cm^−1^): 2542 (O = P-OH), 2228 (O = P-OH), 1259 (P = O), and 1192 (P = O). ^31^P NMR (162 MHz, DMSO-*d_6_*) *δ* = 36.19 ppm. ^1^H NMR (400 MHz, DMSO-*d_6_*) *δ* = 2.64 (d, 1H, *J* = 6.4 Hz), 2.70 (d, 1H, *J* = 6.4 Hz), 5.90–6.03 (m, 1H), 6.64–6.70 (m, 1H), 7.25–7.31 (m, 1H), 7.34–7.43 (m, 2H), 7.60–7.63 (m, 1H), and 7.66–7.68 (m, 2H) ppm. ^13^C NMR (101 MHz, DMSO-*d_6_*) *δ* = 27.5 (d, *J*_P,C_ = 125 Hz), 123.9 (d, *J*_P,C_ = 12.0 Hz), 124.6, 126.9, 128.4, 129.0 (d, *J*_P,C_ = 8.6 Hz), 130.4, 131.3, 131.5, 133.7, 140.8, and 144.7 (d, *J*_P,C_ = 7.4 Hz) ppm. HRMS (ESI) [M–H]^–^: *m/z* calcd for C_15_H_10_O_3_PCl_2_: 338.9745, found 338.9751.

#### 2-Hydroxy-9–(2-nitrophenyl)-3H-benzo[f][1,2]oxaphosphepine 2-oxide (10f)

By following the general procedure, **10f** was prepared from 9-bromo-2-hydroxy-3*H*-benzo[*f*][1,2]oxaphosphepine 2-oxide (**7c**) (200 mg, 0.73 mmol), (2-nitrophenyl)boronic acid (182 mg, 1.09 mmol), K_2_CO_3_ (201 mg, 1.45 mmol), and Pd(dppf)Cl_2_ (106 mg, 0.15 mmol) as a yellowish solid (161 mg, 70%). Mp: 226–228 °C. IR (KBr, cm^−1^): 2359 (O = P-OH), 1229 (P = O), and 1192 (P = O). ^31^P NMR (162 MHz, DMSO-*d_6_*) *δ* = 35.15 ppm. ^1^H NMR (400 MHz, DMSO-*d_6_*) *δ* = 2.66 (d, 1H, *J* = 6.4 Hz), 2.72 (d, 1H, *J* = 6.4 Hz), 5.90–6.03 (m, 1H), 6.65–6.72 (m, 1H), 7.29–7.35 (m, 1H), 7.38–7.45 (m, 2H), 7.86–7.91 (m, 2H), and 8.25–8.31 (m, 2H) ppm. ^13^C NMR (101 MHz, DMSO-*d_6_*) *δ* = 27.5 (d, *J*_P,C_ = 125 Hz), 123.2, 123.9 (d, *J*_P,C_ = 12.0 Hz), 124.7, 128.8, 129.0 (d, *J*_P,C_ = 8.6 Hz), 130.4, 131.0, 131.7, 132.3 (d, *J*_P,C_ = 3.4 Hz), 144.4, 144.8 (d, *J*_P,C_ = 7.4 Hz), and 146.6 ppm. HRMS (ESI) [M–H]^–^: *m/z* calcd for C_15_H_11_NO_5_P: 316.0375, found 316.0385.

### Carbonic anhydrase inhibition assay

The CA-catalysed CO_2_ hydration activity was assayed by using an applied photophysics stopped-flow apparatus as reported in previous papers from our group^22^. All CA isoforms were recombinant proteins, obtained as reported earlier^23–25^.

## Results and discussion

### Chemistry

**Scheme 1.**
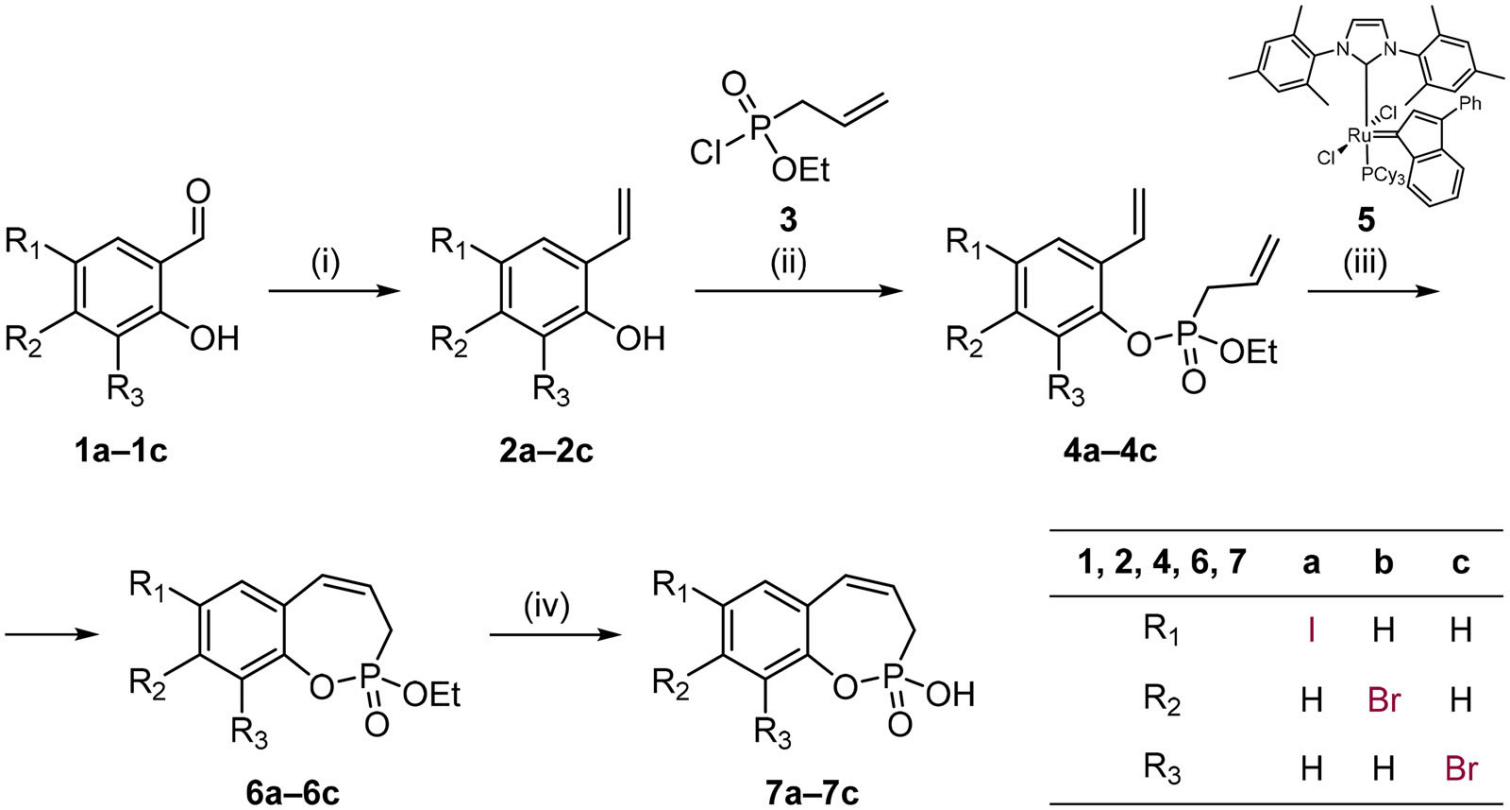
Reagents and conditions: (1) MePPh_3_Br, *t*BuOK, THF, rt, 18 h; (2) NEt_3_, CH_2_Cl_2_, 0 °C to rt, 18 h; (3) **5** (CAS: 250220–36-1), PhMe, 70 °C, 4 h; (iv) TMSBr, CH_2_Cl_2_, rt, 24 h.

Our group recently developed a strategy for the synthesis of simple derivatives of 3*H*-1,2-benzoxaphosphepine 2-oxide from the commercially available salicylaldehydes^19^. This methodology employs a ring-closing metathesis (RCM) reaction as a key step to construct benzo-fused oxaphosphepine ring. Following this synthetic route^19^, we have prepared iodo- and bromo-substituted analogues **7a–c** (Scheme 1). First, halosalicylaldehydes **1a–c** were converted to styrenes **2a–c**. Subsequent phosphorylation and RCM gave the cyclised compounds **6a–c**. Lastly, deprotection was achieved using TMSBr and the target compounds **7a–c** were successfully obtained.

**Scheme 2.**
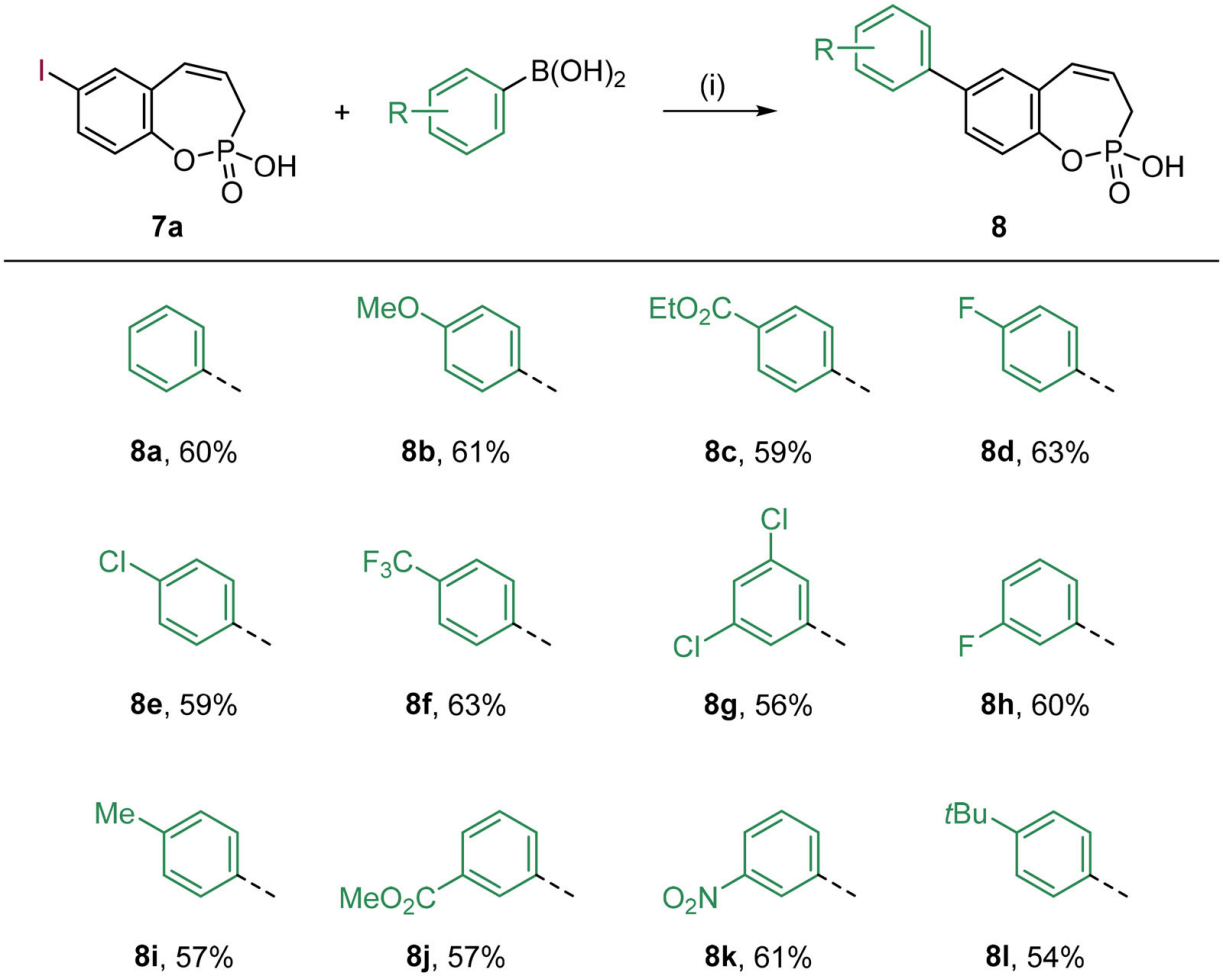
Reagents and conditions: (1) Pd(dppf)Cl_2_, K_2_CO_3_, 1,4-dioxane/H_2_O (5:1), 80 °C, 16 h, 54–63%.

**Scheme 3.**
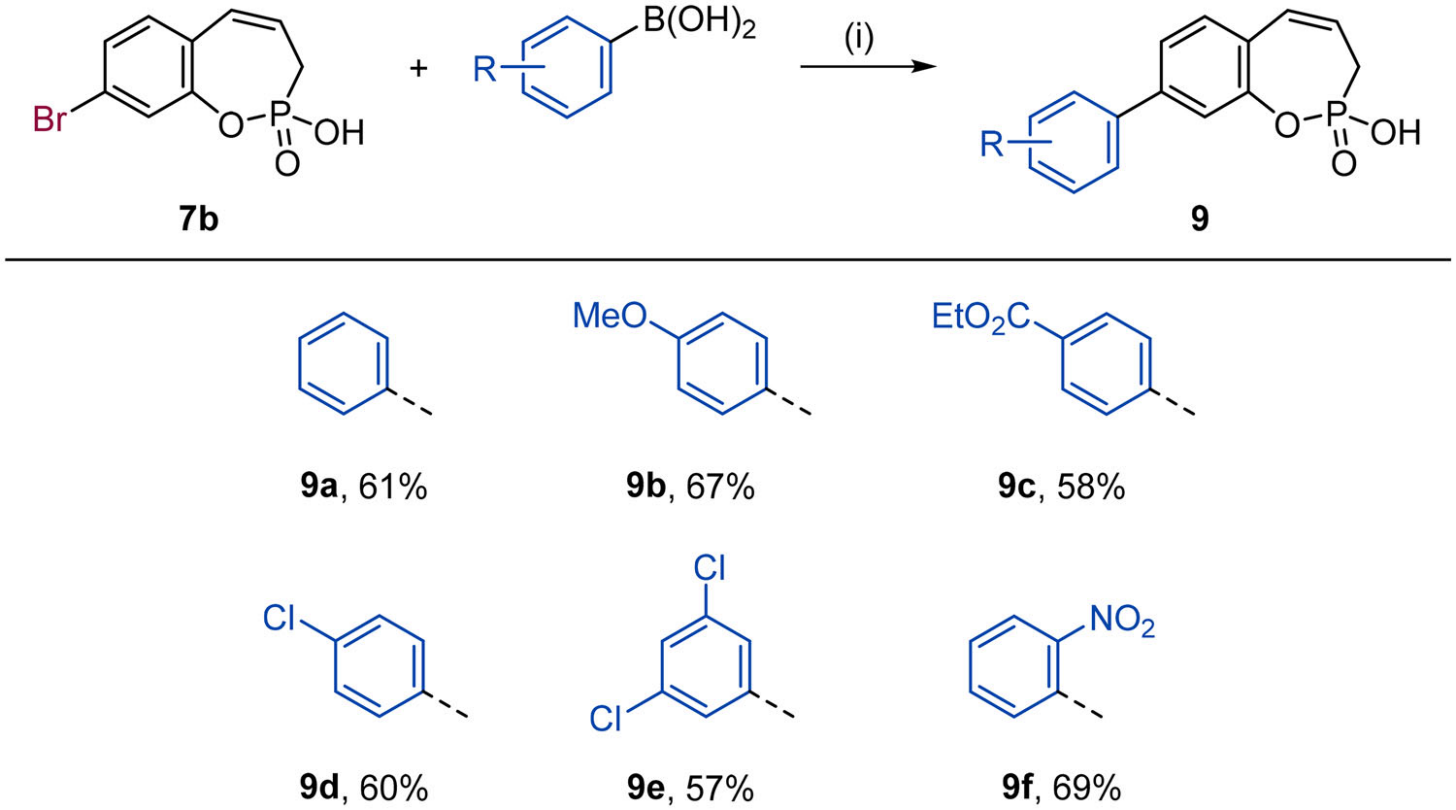
Reagents and conditions: (1) Pd(dppf)Cl_2_, K_2_CO_3_, 1,4-dioxane/H_2_O (5:1), 80 °C, 16 h, 57–69%.

**Scheme 4.**
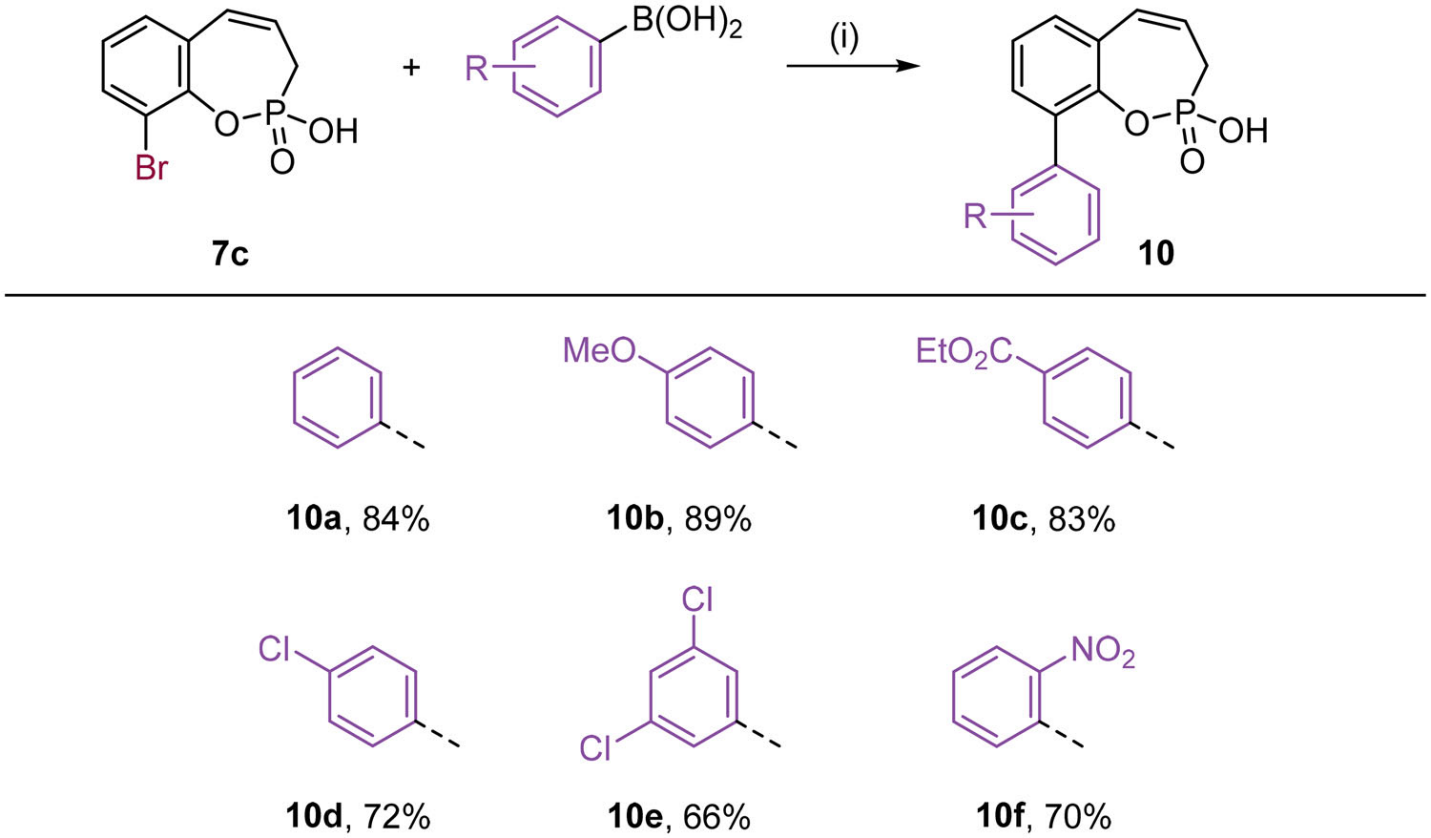
Reagents and conditions: (1) Pd(dppf)Cl_2_, K_2_CO_3_, 1,4-dioxane/H_2_O (5:1), 80 °C, 16 h, 66–89%.

With the halo derivatives **7a–c** in hand, we next proceeded to the Suzuki–Miyaura cross-coupling reaction, employing commercial arylboronic acids. As a result, 7-, 8-, and 9-aryl-substituted benzoxaphosphepine 2-oxides **8–10** were furnished in good to excellent yields (Schemes 2–4). Noteworthy, the electronic nature and substitution patterns of arylboronic acids did not significantly affect isolated yields. However, the coupling reactions occurring at position 9 of benzoxaphosphepine 2-oxide core displayed higher efficacy in yields, compared to positions 7 and 8.

### Carbonic anhydrase inhibition

The newly synthesised compounds **7–10** were investigated for their CA inhibition activity against four pharmacologically relevant human CA isoforms—the ubiquitous cytosolic CA I and II as well as the cancer-associated CA IX and XII. In this study, CA I and CA II are considered off-target isoforms, that were tested in order to explore the selectivity of inhibitors towards the CA IX and CA XII isoforms. The clinically utilised acetazolamide (AAZ) was used as a reference drug. The following structure-activity relationship (SAR) can be deduced from the inhibition data reported in Table 1:

**Table 1.** Inhibition data of compounds **7–10** and the standard inhibitor acetazolamide (AAZ) against human CA isoforms I, II, IX and XII by the stopped-flow CO_2_ hydrase assay. 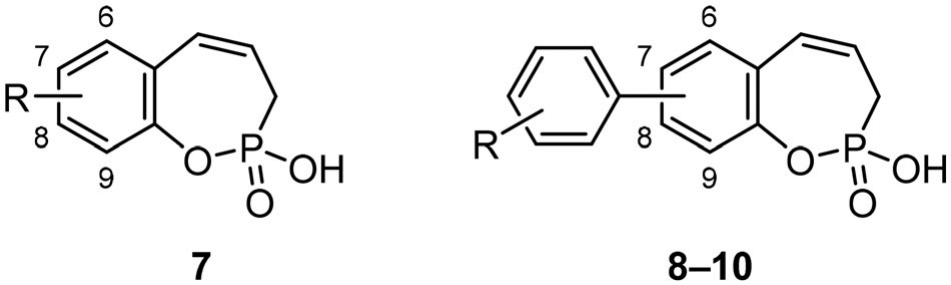
.

Cmpd	Substitution position (7 / 8 / 9)	R	*K*_I_ (µM)^a^^,^^b^			
			CA I	CA II	CA IX	CA XII
**7a** ^19^	7	I	>100	>100	0.88	0.68
**8a**	7	H	>100	>100	0.77	0.95
**8b**	7	4-OMe	>100	>100	4.6	1.7
**8c**	7	4-CO_2_Et	>100	>100	6.0	6.7
**8d**	7	4-F	>100	>100	0.86	0.25
**8e**	7	4-Cl	>100	>100	8.6	1.1
**8f**	7	4-CF_3_	>100	>100	3.7	0.59
**8g**	7	3,5-diCl	>100	>100	7.3	4.2
**8h**	7	3-F	>100	>100	0.63	0.56
**8i**	7	4-Me	>100	>100	1.5	0.94
**8j**	7	3-CO_2_Me	>100	>100	9.5	1.5
**8k**	7	3-NO_2_	>100	>100	7.6	0.64
**8l**	7	4-*t*Bu	>100	>100	4.9	0.97
**7b**	8	Br	>100	>100	3.4	1.5
**9a**	8	H	>100	>100	1.8	2.7
**9b**	8	4-OMe	>100	>100	2.1	5.6
**9c**	8	4-CO_2_Et	>100	>100	10.2	3.8
**9d**	8	4-Cl	>100	>100	5.0	7.1
**9e**	8	3,5-diCl	>100	>100	0.98	0.84
**9f**	8	2-NO_2_	>100	>100	12.9	0.67
**7c** ^19^	9	Br	>100	>100	2.5	1.8
**10a**	9	H	>100	>100	16.5	25.5
**10b**	9	4-OMe	>100	>100	39.4	52.4
**10c**	9	4-CO_2_Et	>100	>100	55.3	>100
**10d**	9	4-Cl	>100	>100	48.9	65.3
**10e**	9	3,5-diCl	>100	>100	22.4	28.2
**10f**	9	4-NO_2_	>100	>100	38.4	35.1
**AAZ**	–		0.25	0.012	0.025	0.006

^a^ Values are mean from three different assays using the stopped-flow technique (errors were in the range of ± 5–10% of the reported values).

^b^ Incubation time: 6 h.

- Equally, as previously reported simple derivatives of benzoxaphosphepine 2-oxide^19^, all aryl derivatives **8–10** reported here, as well as **7b**, showed no inhibitory activity towards the off-target CA isoforms I and II (*K*_I_ > 100 µM). In the context of cancer treatment with CA inhibitors, this is a desirable feature to prevent possible side effects, since CA I and CA II isoforms are found in many tissues throughout the body^6^. The standard drug AAZ has a very good affinity for CA I and CA II.
- The cancer-associated CA IX isoform was inhibited by all of the tested compounds; however, 9-aryl-substituted derivatives **10** were weak inhibitors, with *K*_I_ values ranging from 16.5 to 55.3 µM. In general, 7- and 8-aryl-substituted compounds **8** and **9** were much more effective inhibitors with good or moderate activity. The 7-aryl derivative with the 3-fluoro substituent **8h** emerged as the most potent CA IX inhibitor with *K*_I_ = 0.63 µM. The inclusion of –CO_2_R, –NO_2_ or –Cl substituent typically resulted in decreased inhibitory activity against CA IX.
- Similarly, another cancer-associated isoform CA XII was inhibited by 7-aryl and 8-aryl derivatives **8** and **9**, whereas 9-aryl-substituted derivatives **10** displayed weak or no inhibitory activity (*K*_I_: 25.5–65.3 µM for **10a,b,d–f** and *K*_I_ > 100 µM for **10c** that bears the 4-CO_2_Et substituent). Only the 9-bromo derivative **7c** had a moderate inhibition potency against both CA IX and XII. The 7-aryl derivative with the 4-fluoro substituent **8d** was the most effective inhibitor against CA XII with *K*_I_ = 0.25 µM. Other fluorine-containing compounds also exhibited good activity against CA XII (*K*_I_ = 0.56 µM for **8h**; *K*_I_ = 0.59 µM for **8f**). In contrast to CA IX inhibition profile, 7- and 8-aryl derivatives with the nitro group showed good inhibition of CA XII (*K*_I_ = 0.64 µM for **8k**; *K*_I_ = 0.67 µM for **9f**).

Collectedly, benzoxaphosphepine 2-oxide derivatives that are substituted with aryl groups in positions 7 or 8 displayed superior inhibition efficiency of CA IX and XII as compared to the 9-aryl-substituted derivatives. In comparison with the standard drug AAZ, which is a highly effective inhibitor of all the four CA isoforms considered in this study, the analogues **7–10** were less effective as CA IX and XII inhibitors. However, benzoxaphosphepine 2-oxide derivatives showed desirable selectivity as none of them inhibited the off-target CA I and CA II. The most potent inhibitors of both cancer-associated CA IX and CA XII were fluorine-containing compounds **8d** and **8h**.

It is worthwhile to underline here that a 6-h incubation of the enzyme and each compound **7–10** solutions is essential. When the incubation period of 15 min was used for assaying the inhibition, as generally done for the other types of CA inhibitors, only a weak inhibition was observed (data not shown). Furthermore, it appears to be the case with coumarins and related bioisosteres^9–18^. These compounds were shown to act as prodrug inhibitors, being hydrolysed within the CA active site to the corresponding acids; subsequently, the obtained hydrolysis products bind within the enzyme active site cavity^10^^,^^12^^,^^14a^. By considering aforementioned points, we assume that benzoxaphosphepine 2-oxides are likely to undergo the CA-mediated hydrolysis of oxaphosphepine ring with formation of phosphonic acid derivatives that act as CA inhibitors.

## Conclusions

3*H*-1,2-Benzoxaphosphepine 2-oxides represent a novel chemotype acting as isoform-selective CA inhibitors. In this paper, we have expanded the chemical space around the benzoxaphosphepine scaffold by synthesising aryl derivatives. The latter were evaluated for their inhibitory activity against CA I, II, IX and XII. Most of the compounds tested manifested promising potency in inhibiting the cancer-associated CA isoforms IX and XII. Furthermore, none of the target compounds showed significant inhibition of the cytosolic CA I and II. The SAR studies indicated that 7- and 8-substituted aryl derivatives of 3*H*-1,2-benzoxaphosphepine 2-oxide were considerately more active CA IX/XII inhibitors than the 9-aryl derivatives. The introduction of aryl groups at the 9th position of the scaffold resulted in decreased potency. Among all the tested compounds, derivatives **8d,h** with the fluoro-substituted aryl groups demonstrated the highest inhibitory activity against CA IX/XII, with *K*_I_ values in the sub-micromolar range. Taking into account the efficiency and significant selectivity of these novel molecules, further development and evaluation will be pursued.

## Supplementary Material

Supplemental Material

## References

[1] Sung H, Ferlay J, Siegel RL, Laversanne M, Soerjomataram I, Jemal A, Bray F. Global cancer statistics 2020: GLOBOCAN estimates of incidence and mortality worldwide for 36 cancers in 185 countries. CA Cancer J Clin. 2021;71(3):209–249. 33538338 10.3322/caac.21660

[2] Long J, Ji Z, Yuan P, Long T, Liu K, Li J, Cheng L. Nut consumption and risk of cancer: a meta-analysis of prospective studies. Cancer Epidemiol Biomarkers Prev. 2020;29(3):565–573. 32041895 10.1158/1055-9965.EPI-19-1167

[3] (a) Neri D, Supuran CT. Interfering with pH regulation in tumours as a therapeutic strategy. Nat Rev Drug Discov. 2011;10(10):767–777. 21921921 10.1038/nrd3554

[4] (a) Robertson N, Potter C, Harris AL. Role of carbonic anhydrase IX in human tumor cell growth, survival, and invasion. Cancer Res. 2004;64(17):6160–6165. 15342400 10.1158/0008-5472.CAN-03-2224

[5] (a) Hewett-Emmett D, Tashian RE. Functional diversity, conservation, and convergence in the evolution of the alpha-, beta-, and gamma-carbonic anhydrase gene families. Mol Phylogenet Evol. 1996;5(1):50–77. 8673298 10.1006/mpev.1996.0006

[6] (a) Supuran CT. Structure and function of carbonic anhydrases. Biochem J. 2016;473(14):2023–2032. 27407171 10.1042/BCJ20160115

[7] Kumar S, Rulhania S, Jaswal S, Monga V. Recent advances in the medicinal chemistry of carbonic anhydrase inhibitors. Eur J Med Chem. 2021;209:112923. 33121862 10.1016/j.ejmech.2020.112923

[8] (a)Alterio V, Di Fiore A, D'Ambrosio K, Supuran CT, De Simone G. Multiple binding modes of inhibitors to carbonic anhydrases: How to design specific drugs targeting 15 different isoforms? Chem Rev. 2012;112(8):4421–4468. 22607219 10.1021/cr200176r

[9] Žalubovskis R. In a search for selective inhibitors of carbonic anhydrases: coumarin and its bioisosteres: synthesis and derivatization. Chem Heterocycl Comp. 2015;51(7):607–612.

[10] (a) Maresca A, Temperini C, Vu H, Pham NB, Poulsen S-A, Scozzafava A, Quinn RJ, Supuran CT. Non-zinc mediated inhibition of carbonic anhydrases: coumarins are a new class of suicide inhibitors. J Am Chem Soc. 2009;131(8):3057–3062. 19206230 10.1021/ja809683v

[11] (a) Bonneau A, Maresca A, Winum J-Y, Supuran CT. Metronidazole-coumarin conjugates and 3-cyano-7-hydroxy-coumarin act as isoform-selective carbonic anhydrase inhibitors. J Enzyme Inhib Med Chem. 2013;28(2):397–401. 22299576 10.3109/14756366.2011.650692

[12] Maresca A, Temperini C, Pochet L, Masereel B, Scozzafava A, Supuran CT. Deciphering the mechanism of carbonic anhydrase inhibition with coumarins and thiocoumarins. J Med Chem. 2010;53(1):335–344. 19911821 10.1021/jm901287j

[13] Onyılmaz M, Koca M, Bonardi A, Degirmenci M, Supuran CT. Isocoumarins: a new class of selective carbonic anhydrase IX and XII inhibitors. J Enzyme Inhib Med Chem. 2022;37(1):743–748. 35188025 10.1080/14756366.2022.2041630 PMC8865125

[14] (a) Tars K, Vullo D, Kazaks A, Leitans J, Lends A, Grandane A, Zalubovskis R, Scozzafava A, Supuran CT. Sulfocoumarins (1,2-benzoxathiine-2,2-dioxides): a class of potent and isoform-selective inhibitors of tumor-associated carbonic anhydrases. J Med Chem. 2013;56(1):293–300. 23241068 10.1021/jm301625s

[15] (a) Tanc M, Carta F, Bozdag M, Scozzafava A, Supuran CT. 7-Substituted-sulfocoumarins are isoform-selective, potent carbonic anhydrase II inhibitors. Bioorg Med Chem. 2013;21(15):4502–4510. 23769167 10.1016/j.bmc.2013.05.032

[16] (a) Grandane A, Tanc M, Zalubovskis R, Supuran CT. Synthesis of 6-tetrazolyl-substituted sulfocoumarins acting as highly potent and selective inhibitors of the tumor-associated carbonic anhydrase isoforms IX and XII. Bioorg Med Chem. 2014;22(5):1522–1528. 24513186 10.1016/j.bmc.2014.01.043

[17] Podolski-Renić A, Dinić J, Stanković T, Jovanović M, Ramović A, Pustenko A, Žalubovskis R, Pešić M. Sulfocoumarins, specific carbonic anhydrase IX and XII inhibitors, interact with cancer multidrug resistant phenotype through pH regulation and reverse P-glycoprotein mediated resistance. Eur J Pharm Sci. 2019;138:105012. 31330259 10.1016/j.ejps.2019.105012

[18] (a) Pustenko A, Stepanovs D, Žalubovskis R, Vullo D, Kazaks A, Leitans J, Tars K, Supuran CT. 3*H*-1,2-benzoxathiepine 2,2-dioxides: a new class of isoform-selective carbonic anhydrase inhibitors. J Enzyme Inhib Med Chem. 2017;32(1):767–775. 28537099 10.1080/14756366.2017.1316720 PMC6445229

[19] Pustenko A, Balašova A, Nocentini A, Supuran CT, Žalubovskis R. 3*H*-1,2-Benzoxaphosphepine 2-oxides as selective inhibitors of carbonic anhydrase IX and XII. J Enzyme Inhib Med Chem. 2023;38(1):216–224. 36377338 10.1080/14756366.2022.2143496 PMC9668280

[20] (a) Rodriguez JB, Gallo-Rodriguez C. The role of the phosphorus atom in drug design. ChemMedChem. 2019;14:190–216. 30536636 10.1002/cmdc.201800693

[21] (a) Bonnac L, Innocenti A, Winum J-Y, Casini A, Montero J-L, Scozzafava A, Barragan V, Supuran CT. Carbonic anhydrase inhibitors: aliphatic N-phosphorylated sulfamates – a novel zinc-anchoring group leading to nanomolar inhibitors. J Enzyme Inhib Med Chem. 2004;19(3):275–278. 15500000 10.1080/14756360410001689522

[22] Khalifah RG. The carbon dioxide hydration activity of carbonic anhydrase. I. Stop-flow kinetic studies on the native human isoenzymes B and C. J Biol Chem. 1971;246(8):2561–2573. 4994926

[23] (a) Supuran CT, Ilies MA, Scozzafava A. Carbonic anhydrase inhibitors. Part 29. Interaction of isozymes I, II and IV with benzolamide-like derivatives. Bioorg Med Chem. 1998;33:739–752.

[24] Leitans J, Kazaks A, Balode A, Ivanova J, Zalubovskis R, Supuran CT, Tars K. Efficient expression and crystallization system of cancer-associated carbonic anhydrase isoform IX. J Med Chem. 2015;58(22):9004–9009. 26522624 10.1021/acs.jmedchem.5b01343

[25] (a) Aspatwar A, Parvathaneni NK, Barker H, Anduran E, Supuran CT, Dubois L, Lambin P, Parkkila S, Winum J-W. Design, synthesis, *in vitro* inhibition and toxicological evaluation of human carbonic anhydrases I, II and IX inhibitors in 5-nitroimidazole series. J Enzyme Inhib Med Chem. 2020;35(1):109–117. 31687859 10.1080/14756366.2019.1685510 PMC6844379

