# Supplementary material for: Aryl derivatives of 3H-1,2-benzoxaphosphepine 2-oxides as inhibitors of cancer-related carbonic anhydrase isoforms IX and XII
Source: J Enzyme Inhib Med Chem. 2023 Sep 1;38(1):2249267. doi: 10.1080/14756366.2023.2249267 (PMC10478600; doi:10.1080/14756366.2023.2249267)
Supplement: Supplemental Material [file IENZ_A_2249267_SM1555.pdf]

Supporting information

Aryl derivatives of 3*H*-1,2-benzoxaphosphepine 2-oxides as inhibitors of cancer-related carbonic anhydrase isoforms IX and XII

Anastasija Balašova[a,b], Aleksandrs Pustenko[a], Alessio Nocentini[c], Daniela Vullo[c], Claudiu T. Supuran[c], Raivis Žalubovskis[a,b]*

[a]Latvian Institute of Organic Synthesis, Riga, Latvia

[b]Institute of Technology of Organic Chemistry, Faculty of Materials Science and Applied Chemistry, Riga Technical University, Riga, Latvia

[c]Department of Neurofarba, Section of Pharmaceutical and Nutraceutical Sciences, Florence, Italy

Content

HPLC spectra of representative compounds (**8a**, **8e**, **8g**, **9f**, **10a**, **10b** and **10f**)…………………..S2-S15

HPLC spectra of representative compounds

HPLC of compound **8a**

Empower 3

Apollo_Gr5%_95%UV

SAMPLE INFORMATION

Sample Name:	548-348-NB-183	Acquired By:	System
Sample Type:	Unknown	Sample Set Name:	010823_serviss
Vial:	33	Acq. Method Set:	Serviss_Gr5%
Injection #:	1	Processing Method	Gr_5 %a
Injection Volume:	10.00 ul	Channel Name:	2998 Ch1 254nm@4.8nm
Run Time:	25.0 Minutes	Proc. Chnl. Descr.:	2998 Ch1 254nm@4.8nm
Date Acquired:	01.08.2023 10:07:37 AM EEST		
Date Processed:	01.08.2023 10:38:52 AM EEST		

Apollo C18-13 5um (4.6x150 mm)
15 min Gr. 5-95%ACN +0,1%H3PO4; 5 min Iz. 95%ACN; 2 min Gr. 95-5% ACN; 3 min Iz. 5% ACN.
F=1mL/min. T=40oC.

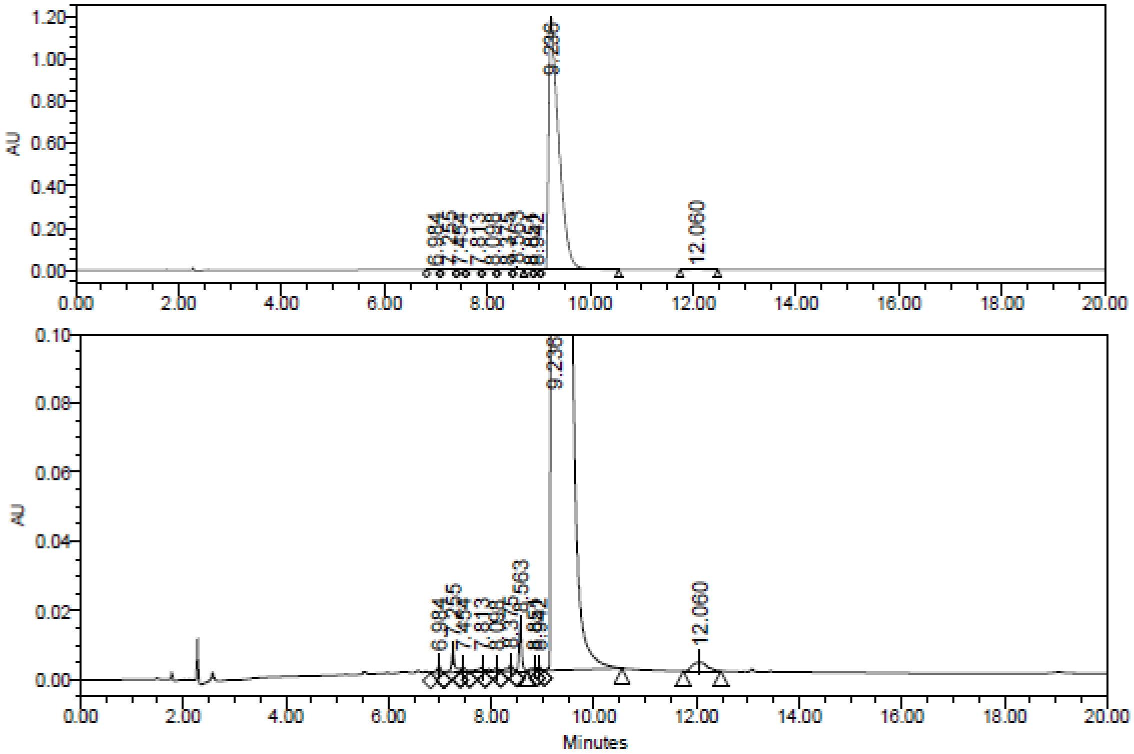

C=0.25 mg/ml (50%ACN_0.1%H3PO4)

	RT	Area	% Area	Height	EP Plate Count	Resolution	Selectivity	Width @ 50%
1	6.984	10675	0.07	1871	51849			0.072
2	7.255	28546	0.17	5617	89106	2.47	1.05	0.057
3	7.454	9348	0.06	1466			1.04	
4	7.813	12056	0.07	1273	22377		1.06	0.123
5	8.098	10316	0.06	1153	8716	1.03	1.05	0.204
6	8.375	15055	0.09	1934	67836	1.17	1.04	0.076
7	8.563	47081	0.29	12502	128524	1.68	1.03	0.056
8	8.851	6619	0.04	1172	25037	1.81	1.04	0.132
9	8.942	4955	0.03	1109	84070	0.53	1.01	0.073
10	9.236	16142016	98.80	1191706	11637	1.27	1.04	0.202
11	12.060	51550	0.32	2637	8565	6.56	1.37	0.307
Sum		16338217.2						

HPLC of compound **8e**

Kinetex_Gr5%_95%UV

SAMPLE INFORMATION

Sample Name:	540-344-NB-184	Acquired By:	System
Sample Type:	Unknown	Sample Set Name:	280723_serviss
Vial:	27	Acq. Method Set:	Gr 5_95%_30min
Injection #:	1	Processing Method	Gr_5 %a
Injection Volume:	10.00 ul	Channel Name:	2998 Ch1 254nm@4.8nm
Run Time:	35.0 Minutes	Proc. Chnl. Descr.:	2998 Ch1 254nm@4.8nm
Date Acquired:	28.07.2023 11:48:51 AM EEST		
Date Processed:	31.07.2023 10:55:45 AM EEST		

Kinetex C 18 5um (4.6x150 mm)
30 min Gr. 5-95%ACN +0,1%H3PO4; 5 min Iz. 95%ACN; 2 min Gr. 95-5% ACN; 3 min Iz. 5% ACN.
F=1mL/min. T=40oC.

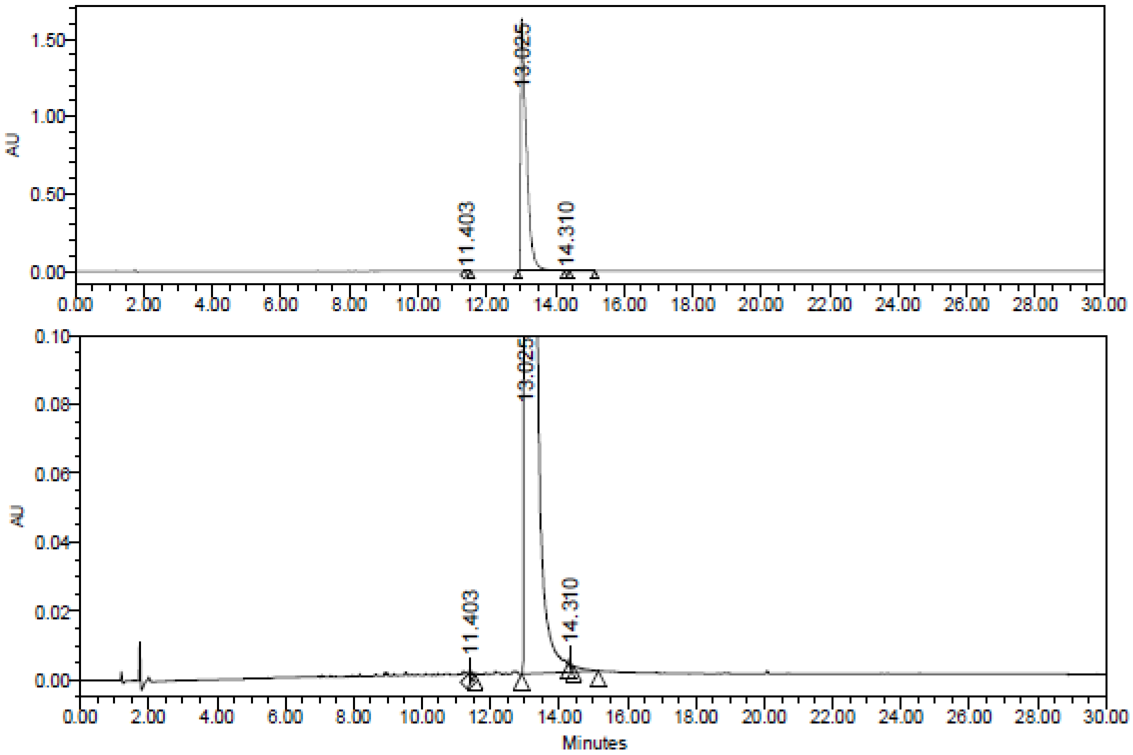

C=0.3 mg/ml (35%ACN_0.1%H3PO4)

	RT	Area	% Area	Height	EP Plate Count	Resolution	Selectivity	Width @ 50%
1	11.403	6823	0.04	1148	71340			0.100
2	13.025	19308996	99.94	1630892	30729	6.95	1.16	0.175
3	14.310	5578	0.03	1951			1.11	
Sum		19321396.7						

HPLC of compound **8g**

Kinetex_Gr5%_95%UV

SAMPLE INFORMATION

Sample Name:	539-343-NB-177	Acquired By:	System
Sample Type:	Unknown	Sample Set Name:	280723_serviss
Vial:	29	Acq. Method Set:	Gr 5_95%_30min
Injection #:	1	Processing Method	Gr_5 %a
Injection Volume:	10.00 ul	Channel Name:	2998 Ch1 254nm@4.8nm
Run Time:	35.0 Minutes	Proc. Chnl. Descr.:	2998 Ch1 254nm@4.8nm
Date Acquired:	28.07.2023 1:00:51 PM EEST		
Date Processed:	31.07.2023 11:14:46 AM EEST		

Kinetex C 18 5um (4.6x150 mm)
30 min Gr. 5-95%ACN +0,1%H3PO4; 5 min Iz. 95%ACN; 2 min Gr. 95-5% ACN; 3 min Iz. 5% ACN.
F=1mL/min. T=40oC.

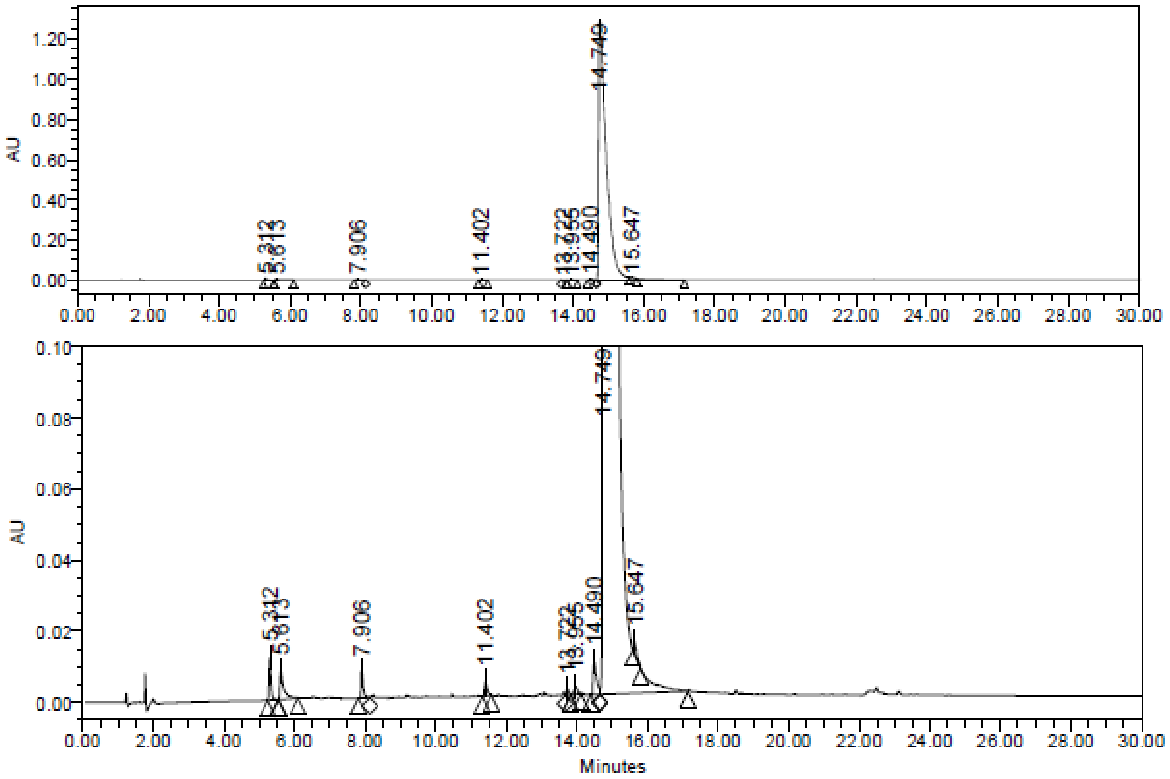

C=0.39 mg/ml (25%ACN_0.1%H3PO4)

	RT	Area	% Area	Height	EP Plate Count	Resolution	Selectivity	Width @ 50%
1	5.312	44860	0.22	12146	55517			0.053
2	5.613	58913	0.29	8118	25353	2.61	1.08	0.083
3	7.906	31506	0.16	7542	104902	19.27	1.57	0.057
4	11.402	22352	0.11	4168	120245	30.58	1.55	0.077
5	13.722	8742	0.04	1753	166061	17.48	1.24	0.079
6	13.955	16961	0.08	2251	88325	1.45	1.02	0.111
7	14.490	62195	0.31	9378	121908	3.03	1.04	0.098
8	14.749	20014342	98.67	1294407	24273	0.95	1.02	0.223
9	15.647	24935	0.12	3911			1.07	
Sum		20284804.9						

HPLC of compound **9f**

Apollo_Gr5%_95%UV

SAMPLE INFORMATION

Sample Name:	553-353-NB-266	Acquired By:	System
Sample Type:	Unknown	Sample Set Name:	010823_serviss
Vial:	38	Acq. Method Set:	Serviss_Gr5%
Injection #:	1	Processing Method	Gr_5 %a
Injection Volume:	10.00 ul	Channel Name:	2998 Ch1 254nm@4.8nm
Run Time:	25.0 Minutes	Proc. Chnl. Descr.:	2998 Ch1 254nm@4.8nm
Date Acquired:	01.08.2023 12:17:26 PM EEST		
Date Processed:	01.08.2023 1:30:01 PM EEST		

Apollo C18-13 5um (4.6x150 mm)
15 min Gr. 5-95%ACN +0,1%H3PO4; 5 min Iz. 95%ACN; 2 min Gr. 95-5% ACN; 3 min Iz. 5% ACN.
F=1mL/min. T=40oC.

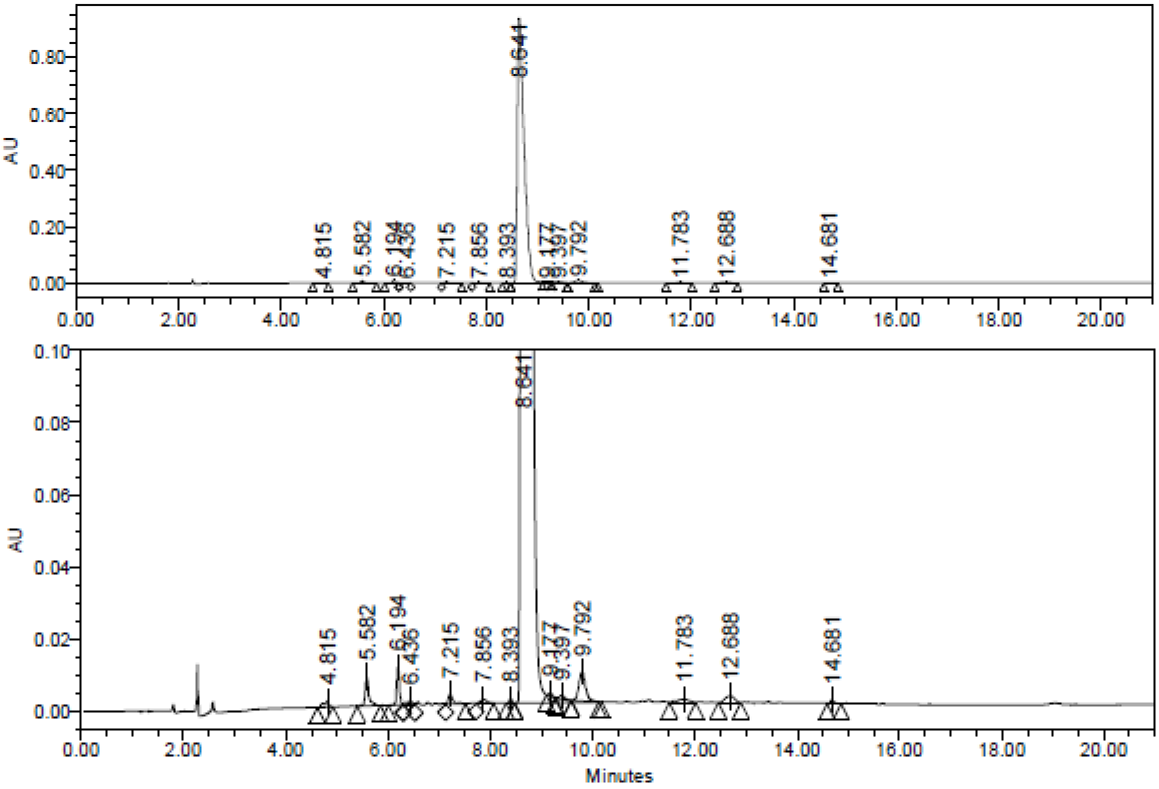

C=0.20 mg/ml (60%ACN_0.1%H3PO4)

	RT	Area	% Area	Height	EP Plate Count	Resolution	Selectivity	Width @ 50%
1	4.815	11607	0.13	1781	6575			0.140
2	5.582	41416	0.47	8101	38702	4.38	1.24	0.067
3	6.194	39151	0.44	10582	69934	5.92	1.15	0.055
4	6.436	7813	0.09	1315	64855	2.50	1.05	0.059
5	7.215	17329	0.20	2777	38959	6.31	1.16	0.086
6	7.856	10644	0.12	1250	24276	3.69	1.11	0.119
7	8.393	4626	0.05	1357	136388	3.68	1.09	0.053
8	8.641	8593726	97.06	934868	20910	1.51	1.04	0.141
9	9.177	4394	0.05	851			1.08	
10	9.397	7482	0.08	995			1.03	
11	9.792	74958	0.85	8116			1.05	
12	11.783	13513	0.15	864	14741		1.24	0.228
13	12.688	22626	0.26	1776	20482	2.44	1.09	0.209
14	14.681	4504	0.05	1031	295093	8.64	1.18	0.064
Sum		8853790.2						

HPLC of compound **10a**

Apollo_Gr5%_95%UV

SAMPLE INFORMATION

Sample Name:	537-341-NB-277	Acquired By:	System
Sample Type:	Unknown	Sample Set Name:	270723_serviss
Vial:	25	Acq. Method Set:	Serviss_Gr5%
Injection #:	1	Processing Method	Gr_5 %
Injection Volume:	10.00 ul	Channel Name:	2998 Ch1 254nm@4.8nm
Run Time:	25.0 Minutes	Proc. Chnl. Descr.:	2998 Ch1 254nm@4.8nm
Date Acquired:	27.07.2023 10:16:47 AM EEST		
Date Processed:	27.07.2023 10:56:45 AM EEST		

Apollo C18-13 5um (4.6x150 mm)
15 min Gr. 5-95%ACN +0,1%H3PO4; 5 min Iz. 95%ACN; 2 min Gr. 95-5% ACN; 3 min Iz. 5% ACN.
F=1mL/min. T=40oC.

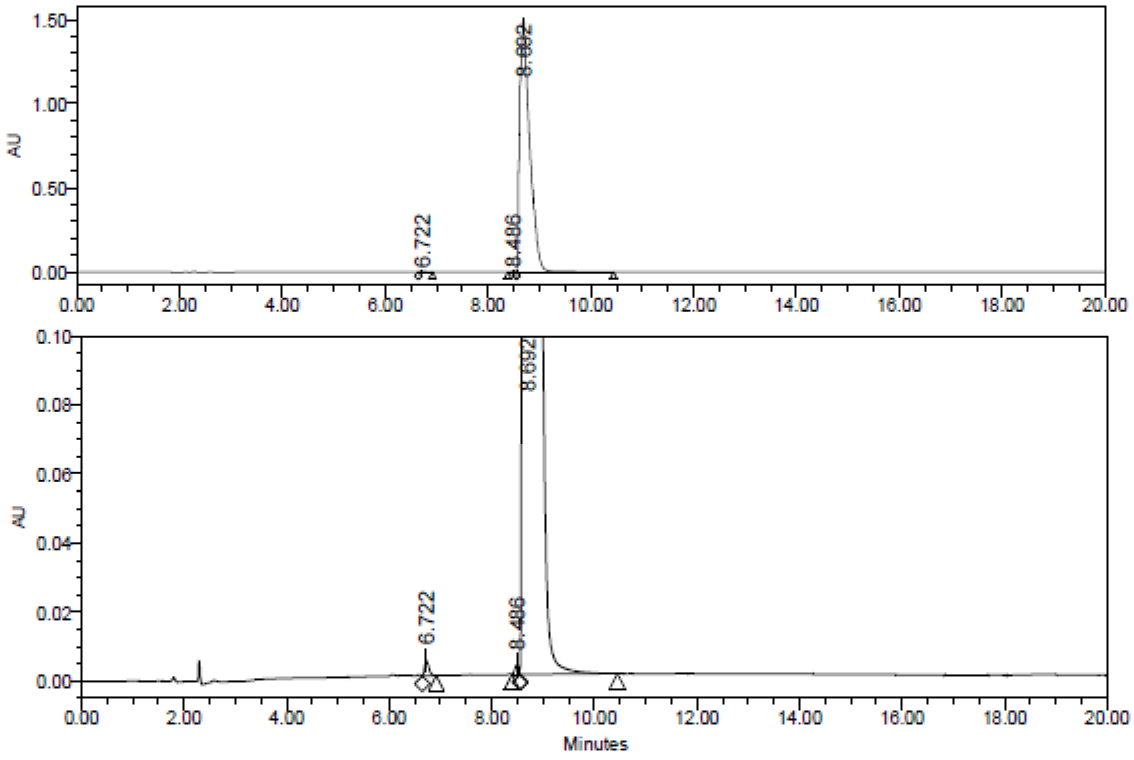

C=0.5 mg/ml (40%ACN_0.1%H3PO4)

	RT	Area	% Area	Height	EP Plate Count	Selectivity	Width @ 50%
1	6.722	25401	0.13	4390	29676		0.092
2	8.486	15315	0.08	2616		1.34	
3	8.692	20085725	99.80	1499070	9325	1.03	0.212
Sum		20126441.6					

HPLC of compound **10b**

Apollo_Gr5%_95%UV

SAMPLE INFORMATION

Sample Name:	551-351-NB-268	Acquired By:	System
Sample Type:	Unknown	Sample Set Name:	010823_serviss
Vial:	36	Acq. Method Set:	Serviss_Gr5%
Injection #:	1	Processing Method	Gr_5 %a
Injection Volume:	10.00 ul	Channel Name:	2998 Ch1 254nm@4.8nm
Run Time:	25.0 Minutes	Proc. Chnl. Descr.:	2998 Ch1 254nm@4.8nm
Date Acquired:	01.08.2023 11:25:25 AM EEST		
Date Processed:	01.08.2023 12:45:11 PM EEST		

Apollo C18-13 5um (4.6x150 mm)
15 min Gr. 5-95%ACN +0,1%H3PO4; 5 min Iz. 95%ACN; 2 min Gr. 95-5% ACN; 3 min Iz. 5% ACN.
F=1mL/min. T=40oC.

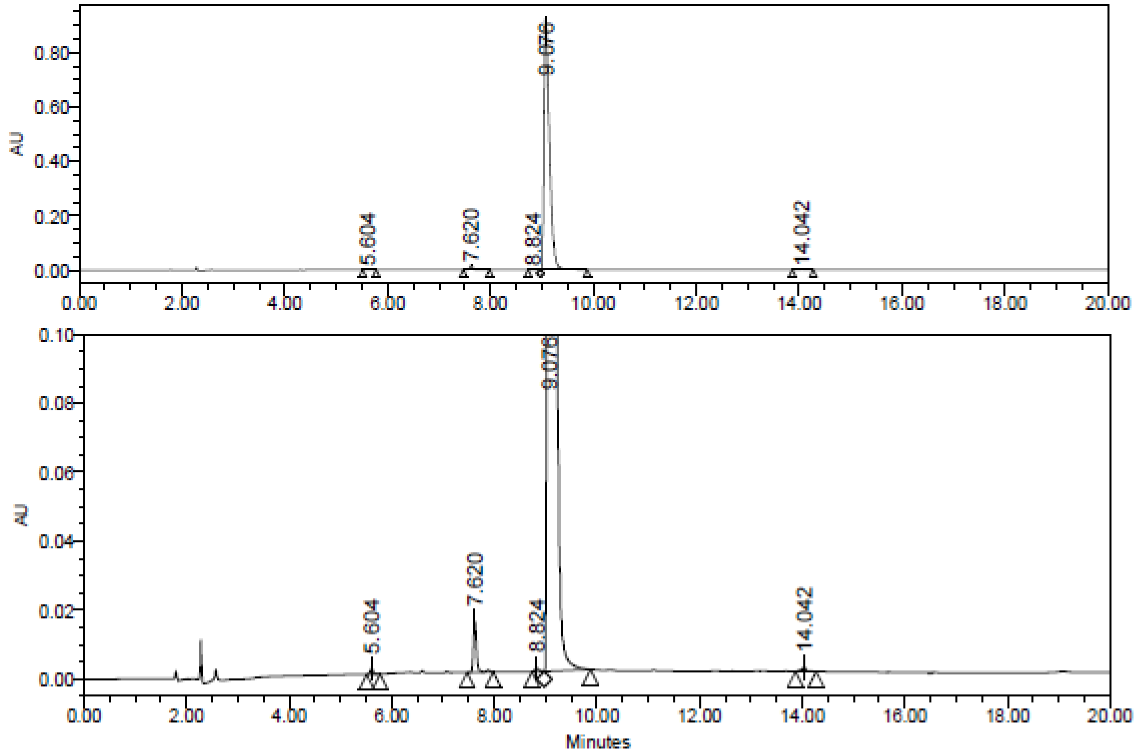

C=0.12 mg/ml (50%ACN_0.1%H3PO4)

	RT	Area	% Area	Height	EP Plate Count	Resolution	Selectivity	Width @ 50%
1	5.604	6550	0.09	1578	45571			0.062
2	7.620	63902	0.92	14755	82616	19.16	1.50	0.062
3	8.824	6291	0.09	1058	63202	9.79	1.20	0.083
4	9.076	6836839	98.77	927423	37192	1.54	1.03	0.111
5	14.042	8103	0.12	1275	198898	31.69	1.66	0.074
Sum		6921685.6						

HPLC of compound **10f**

Apollo_Gr5%_95%UV

SAMPLE INFORMATION

Sample Name:	554-354-NB-297	Acquired By:	System
Sample Type:	Unknown	Sample Set Name:	010823_serviss
Vial:	39	Acq. Method Set:	Serviss_Gr5%
Injection #:	1	Processing Method	Gr_5 %a
Injection Volume:	10.00 ul	Channel Name:	2998 Ch1 254nm@4.8nm
Run Time:	25.0 Minutes	Proc. Chnl. Descr.:	2998 Ch1 254nm@4.8nm
Date Acquired:	01.08.2023 12:43:14 PM EEST		
Date Processed:	01.08.2023 2:08:02 PM EEST		

Apollo C18-13 5um (4.6x150 mm)
15 min Gr. 5-95%ACN +0,1%H3PO4; 5 min Iz. 95%ACN; 2 min Gr. 95-5% ACN; 3 min Iz. 5% ACN.
F=1mL/min. T=40oC.

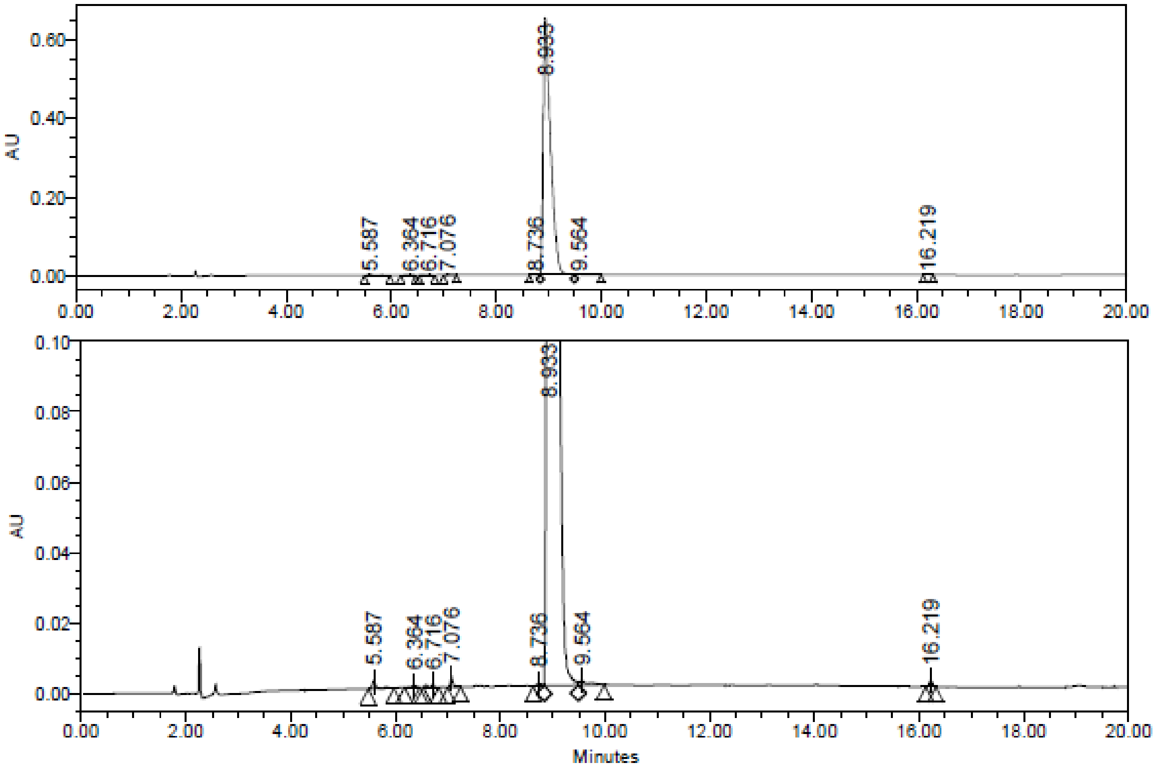

C=0.20 mg/ml (60%ACN_0.1%H3PO4)

	RT	Area	% Area	Height	EP Plate Count	Resolution	Selectivity	Width @ 50%
1	5.587	11440	0.18	2084	38589			0.067
2	6.364	3464	0.05	550	33467	6.16	1.19	0.082
3	6.716	3988	0.06	633	7647	1.58	1.07	0.181
4	7.076	9314	0.14	2493	88135	1.79	1.07	0.056
5	8.736	5020	0.08	762			1.30	
6	8.933	6390333	99.16	652220	19700		1.03	0.150
7	9.564	14692	0.23	1211			1.09	
8	16.219	6519	0.10	1686	396606		1.83	0.061
Sum		6444769.1						
